# Balancing of the mitotic exit network and cell wall integrity signaling governs the development and pathogenicity in *Magnaporthe oryzae*

**DOI:** 10.1371/journal.ppat.1009080

**Published:** 2021-01-07

**Authors:** Wanzhen Feng, Ziyi Yin, Haowen Wu, Peng Liu, Xinyu Liu, Muxing Liu, Rui Yu, Chuyun Gao, Haifeng Zhang, Xiaobo Zheng, Ping Wang, Zhengguang Zhang

**Affiliations:** 1 Department of Plant Pathology, College of Plant Protection, Nanjing Agricultural University, and Key Laboratory of Integrated Management of Crop Diseases and Pests, Ministry of Education, Nanjing, China; 2 The Key Laboratory of Plant Immunity, Nanjing Agricultural University, Nanjing, China; 3 Departments of Microbiology, Immunology, and Parasitology, and Pediatrics, Louisiana State University Health Sciences Center, New Orleans, Louisiana, United States of America; Institute of Microbiology, CHINA

## Abstract

The fungal cell wall plays an essential role in maintaining cell morphology, transmitting external signals, controlling cell growth, and even virulence. Relaxation and irreversible stretching of the cell wall are the prerequisites of cell division and development, but they also inevitably cause cell wall stress. Both Mitotic Exit Network (MEN) and Cell Wall Integrity (CWI) are signaling pathways that govern cell division and cell stress response, respectively, how these pathways cross talk to govern and coordinate cellular growth, development, and pathogenicity remains not fully understood. We have identified MoSep1, MoDbf2, and MoMob1 as the conserved components of MEN from the rice blast fungus *Magnaporthe oryzae*. We have found that blocking cell division results in abnormal CWI signaling. In addition, we discovered that MoSep1 targets MoMkk1, a conserved key MAP kinase of the CWI pathway, through protein phosphorylation that promotes CWI signaling. Moreover, we provided evidence demonstrating that MoSep1-dependent MoMkk1 phosphorylation is essential for balancing cell division with CWI that maintains the dynamic stability required for virulence of the blast fungus.

## Author summary

The cell wall is a relatively rigid structure for supporting the cell shape and against extracellular stresses. However, it also maintains plasticity to cope with cell division, growth, and differentiation. In the rice blast pathogenic fungus *Magnaporthe oryzae*, such differentiation corresponds directly to its virulence. Thus, how to balance the “strong for shaping” with the “malleable for growth and virulence” poses as an important question of both basic- and applied science of significance. We here report that the protein kinase MoSep1 links the Mitotic Exit Network (MEN) to the Cell Wall Integrity (CWI) signaling through the phosphorylation of the CWI MAP kinase kinase MoMkk1. We found that the MoSep1-dependent phosphorylation of MoMkk1 relieves the cell wall stress caused by cell division and that the MEN-CWI-mediated balance of rigid and remodeling of the cell wall is important in the growth, development, and virulence of the blast fungus. Our study provides a new evidence on how the blast fungus adapts to self-generated stress for growth and virulence and it sheds new light on the crosstalk between MEN and CWI signaling.

## Introduction

The cell wall plays a critical role in maintaining cell morphology and progression throughout the cell cycle, and its composition depends on species. It varies in the cell types and developmental stages [1]. In plants, cellulose and pectin are abundant in the primary cell wall that influence the cell wall porosity and extensibility during the cell cycle [2], while lignin found in the secondary cell wall protects the cell from the external stress [3]. In filamentous fungi, α-(1,3) glucan and chitin of the cell wall form a hydrophobic scaffold surrounded by a hydrated matrix of β-glucans and capped by a layer of glycoproteins and α-1,3-glucan [4–5]. The cell wall protects against the osmotic shock or mechanical stresses [6–7], and may also induce the host’s immune response [8]. Thus, the cell wall integrity is critical to its function, including pathogenesis [9].

The Cell Wall Integrity (CWI) pathway is one of the most pivotal signal transmission mechanisms in response to cell wall stresses in the budding yeast *Saccharomyces cerevisiae* and pathogenic fungi, such as the rice blast fungus *Magnaporthe oryzae* [1,10]. The yeast CWI pathway mainly consists of a conserved Mitogen-Activated Protein (MAP) kinase kinase kinase (Bck1), a redundant MAP kinase kinase (Mkk1 and Mkk2), and a MAP kinase (Mpk1/Slt2) [11–13]. The disruption of the MAP kinase pathway components results in various cell lysis [1,14]. In *M*. *oryzae*, the MAP kinase pathway is composed of MoMck1 (the Bck1 homolog), MoMkk1 (the Mkk1/2 homolog), and MoMps1 (the Mpk1/Slt2 homolog) that play an essential role in aerial hyphae morphogenesis, appressorium formation, and pathogenicity [10,14–15]. In addition, recent studies have found that other kinases, such as autophagy-related MoAtg1, could also phosphorylate MoMkk1 in response to the endoplasmic reticulum (ER) stress [16]. This study suggested the importance and the multitude of regulatory mechanisms by CWI signaling.

*M*. *oryzae* produces a specialized infection structure called appressorium to initiate the infection [17–18]. Appressorium formation requires not only the recognition of physical signals such as hardness and hydrophobicity but also proper cell cycle [19–21]. The normal conidium contains three single-nucleated cells, and the cells then germinate to produce the germ tubes, each containing a newly produced nucleus. This new nucleus will enter the appressorium while the nuclei remaining in the conidium undergo degradation. The nucleus in the appressorium will migrate to the invading peg (nail) and replicate multiple rounds during the infectious hypha (IH) expansion [22–27]. Therefore, the infection-related process is tightly linked to the cell division in *M*. *oryzae*.

In yeast, the mitotic exit network (MEN) is required for normal mitosis during the budding process. The small GTPase Tem1 targets the spindle positioning checkpoint (SPOC) to regulate the MEN activity whereas Tem1 itself is regulated by the GTPase-activating protein (GAP) Bub2-Bfa1 [28]. During the late anaphase, Tem1 activates the protein kinase Cdc15, activating the downstream kinase Dbf2 associated with its activating subunit, Mob1 [29]. Previous studies in *M*. *oryzae* showed that the deletion of MoBub2 (the Bub2 homologs) affects the switch from apical to polar growth due to the shortened G1 phase [22]. Also, the expression of a temperature-sensitive allele of Cdc15 homolog resulted in increased septation and nuclear division in the germ tubes and the defects in appressorium formation and plant infection [23]. These studies suggested conserved mechanisms may present in MEN and its regulation between the yeast and the blast fungus.

During incessant cell division accompanying vegetative growth, spore germination, and infection-related development, cells may rely on the CWI pathway to relieve the stress associated with the cell wall’s continuous stretching and contracting [10,30]. There have been recent attempts to connect cell division with CWI [9,31–32]. We here report identifying a novel link between the cell cycle control and CWI signaling that underlies the growth and pathogenicity of the rice blast fungus.

## Results

### The balance between mitosis and CWI is vital for the vegetative and infectious growth of *M*. *oryzae*

**Fig 1:**
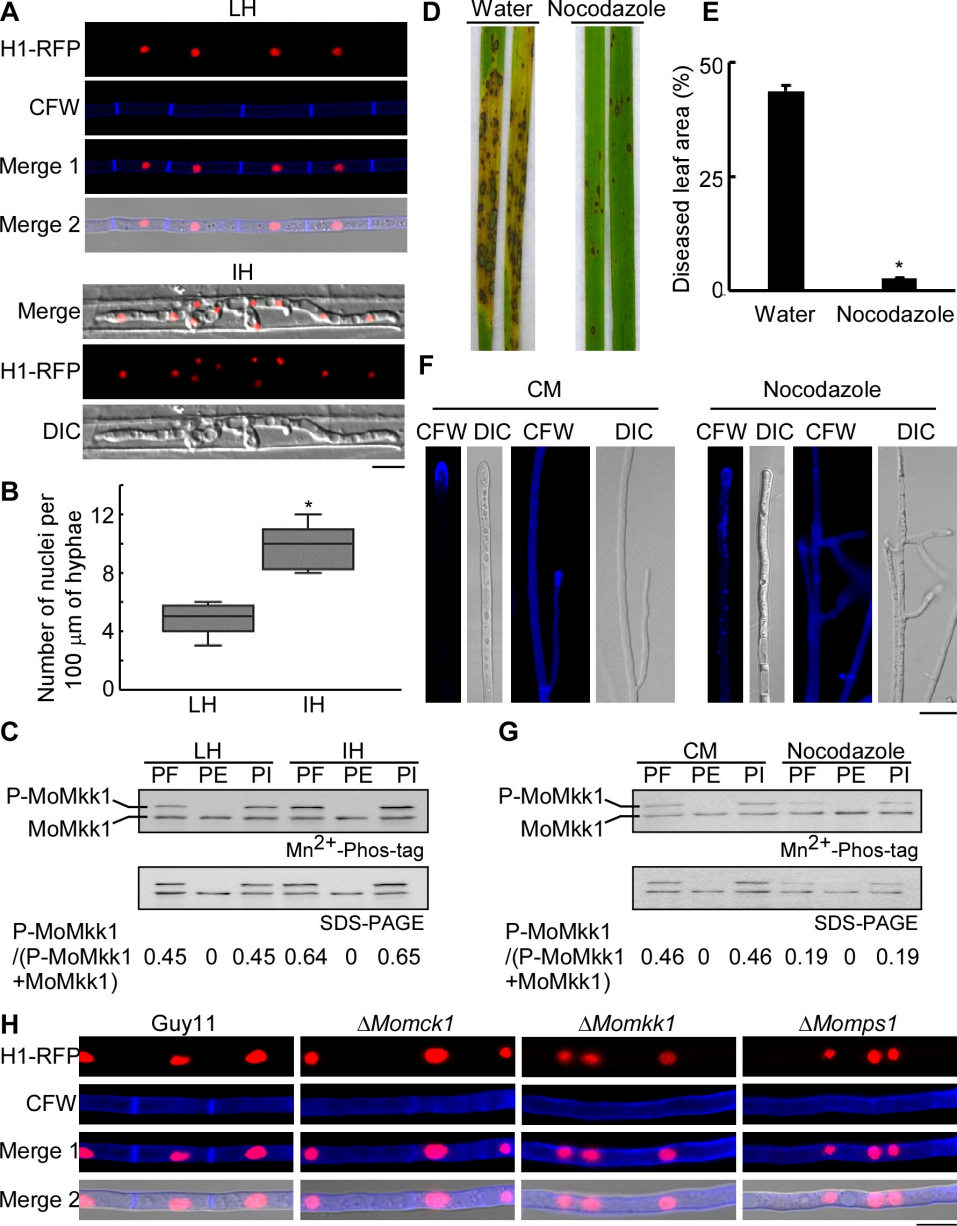
The balance of mitosis and cell wall integrity (CWI) is vital for vegetative and infectious growth of *M*. *oryzae*. (A) Liquid cultured hyphae (LH) and infectious hyphae (IH) of the Guy11 transformant expressing H1-RFP were examined by epifluorescence microscopy. Bar, 10 μm. (B) Statistical analysis of the number of nuclei per 100 μm of hyphae of (A). One hundred hyphae were counted for each type and the experiment was repeated three times. Error bars represent the standard deviations. Asterisks indicate statistical significance according to a Student’s test (p<0.01). (C) Phosphorylation analysis of MoMkk1 in LH and IH. MoMkk1-GFP proteins were extracted in the presence of PMSF (PF), phosphatase (PE), as well as phosphatase inhibitors (PI), respectively, and then detected by the anti-GFP antibody. MoMkk1 phosphorylation was estimated by calculating the amount of phosphorylated-MoMkk1 (P-MoMkk1) compared to the total amount of MoMkk1 (the numbers underneath the blot). (D) Pathogenicity test in rice. The spore suspension of Guy11 was divided into two parts, one was treated with Nocodazole, and another without. Two-week-old rice seedlings were inoculated with these conidial suspensions treated with or without Nocodazole, and photographed at 7 days post-inoculation (dpi). (E) Diseased leaf area analysis of (D). Data were presented as a bar chart showing the percentage of lesion areas. Error bars represented the standard deviations from three independent experiments. Asterisks indicate statistical significance according to a Student’s test (p<0.01). (F) Wild-type hyphae treated with or without Nocodazole were stained with CFW and observed by epifluorescence microscopy. Bar, 10 μm. (G) Phosphorylation analysis of MoMkk1 in Guy11 treated with or without Nocodazole (the steps are similar to C). (H) Hyphae of Guy11 and the Δ*Momck1*, Δ*Momkk1*, Δ*Momps1* mutants expressing the H1-RFP construct were stained with CFW and examined by epifluorescence microscopy. Bar, 10 μm.

To examine the link between cell division and CWI signaling, we co-introduced a histone H1-red fluorescent protein (RFP) and a MoMkk1-green fluorescent protein (GFP) gene fusion constructs into the wild-type strain Guy11. When stained with Calcofluor White (CFW), we found that hyphae grown in liquid culture exhibit a uniformly linear structure with one nucleus per cell. The septa are evenly distributed, in contrast to the infectious hyphae that are swollen (Fig 1A). The hyphae grown in liquid culture contained about 4–6 nuclei per 100 μm hyphae, in contrast to 8–11 nuclei in the infectious hyphae (Fig 1B). This indicated that the rate of cell division under vegetative and infectious growth was different. To investigate the role of CWI in the varied distribution of nuclei, we estimated MoMkk1 phosphorylation levels by Mn^2+^-Phos-tag SDS-PAGE. We found that the density of the phosphorylated-MoMkk1-GFP (P-MoMkk1) band is higher than that of MoMkk1-GFP (MoMkk1) in the presence of a phosphatase inhibitor, but not when the phosphatase was added. The P-MoMkk1 band from liquid-cultured hyphae was significantly weaker than that from the infectious hyphae (Fig 1C).

To further explore the connection between CWI signaling and mitosis, we sprayed the conidial suspension of Guy11 treated by Nocodazole that inhibits mitosis onto 14 days old rice seedlings. The pathogenicity decreased significantly compared with the untreated conidial suspension (Fig 1D and 1E). We then added Nocodazole to the liquid culture and found that the normally even distribution of chitin is altered (Fig 1F). Nocodazole also caused abnormal hyphal branching in comparison to untreated hyphae with angled but forward branching (Fig 1F). Consistent with the fluorescence observation results, the P-MoMkk1 level with Nocodazole treatment was much weaker than that without (Fig 1G). We introduced H1-RFP construct into Δ*Momck1*, Δ*Momkk1*, and Δ*Momps1* mutants, respectively. Following CFW staining, we found that the hyphae contain multiple nuclei in each cell which shows that cell division is disordered when the cell wall integrity pathway is blocked (Fig 1H). These results indicated that there is a close relationship between CWI and cell division and that the dynamic balance between them is important for the normal vegetative growth of the fungus.

### MoMkk1 interacts with the MEN kinase MoSep1

**Fig 2:**
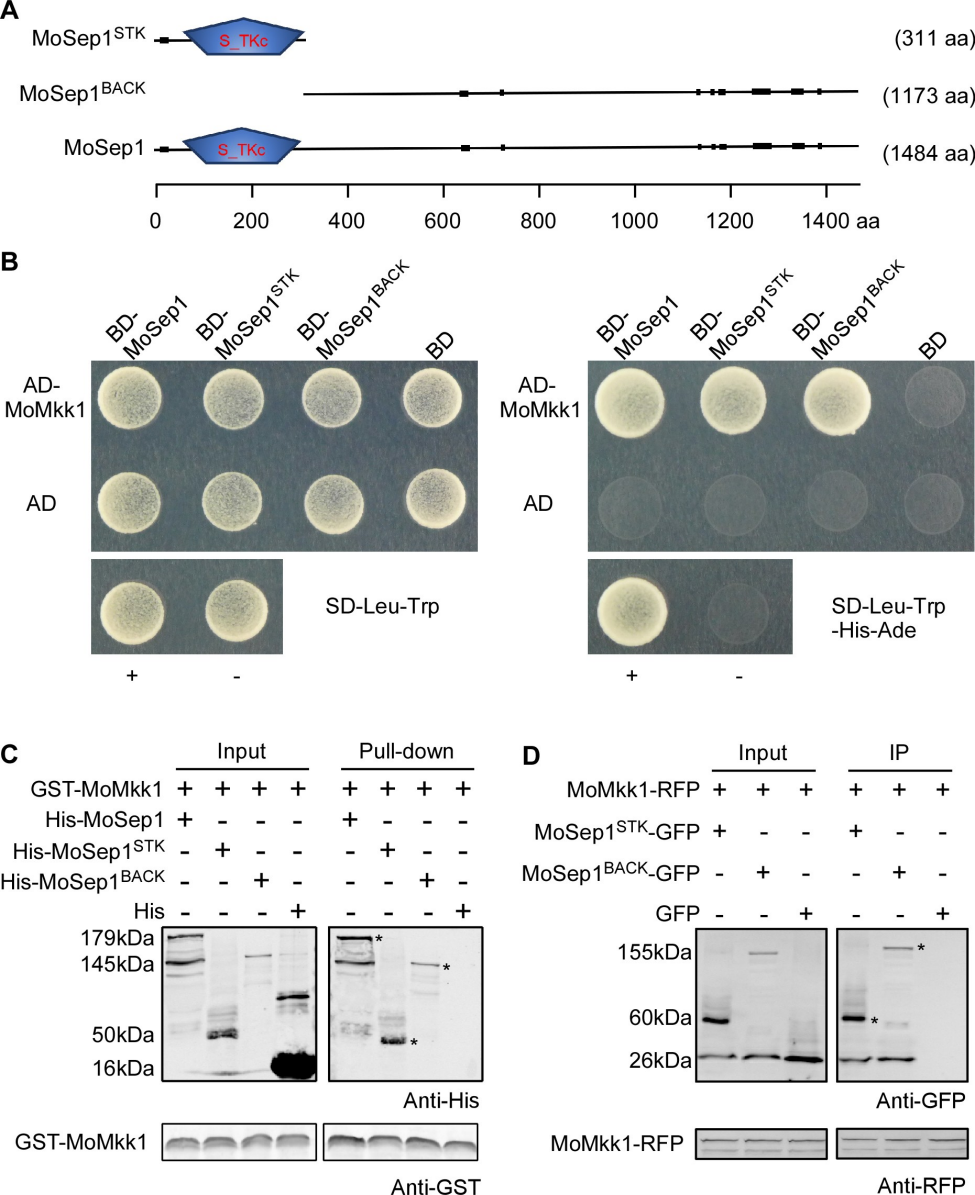
The Mitosis Exit Network (MEN) kinase MoSep1 interacts with MoMkk1. (A) Schematic representation of MoSep1 and its two segments MoSep1^STK^ and MoSep1^BACK^. S_TKc domain was predicted using the SMART program (http//smart.embl-heidelberg.de/). aa, amino acid. (B) Yeast two-hybrid analysis of the interaction between MoMkk1 and MoSep1. MoMkk1 was inserted into vector pGADT7, two segments of MoSep1 (MoSep1^STK^ and MoSep1^BACK^), and MoSep1 full-length were inserted into pGBKT7. AD-MoMkk1 and BD-MoSep1, AD-MoMkk1 and BD-MoSep1^STK^, AD-MoMkk1, and BD-MoSep1^BACK^ were co-introduced into yeast AH109 strain, respectively, and then incubated on SD-Leu-Trp (as control) and SD-Leu-Trp-His-Ade (for selection) for 5 days. (C) *In vitro* pull-down assay of GST-MoMkk1 and His-MoSep1, His-MoSep1^STK^, His- MoSep1^BACK^. Recombinant GST-MoMkk1 bound to glutathione Sepharose beads was incubated with the bacterial cell lysate containing His-MoSep1, His-MoSep1^STK^, His-MoSep1^BACK^, respectively. Eluted protein was analyzed by immunoblot (IB) with anti-His and anti-GST antibodies. GST, glutathione transferase. His, histidine. (D) Co-IP assay for the interaction between MoMkk1 and MoSep1. Plasmids of MoMkk1-RFP and MoSep1^STK^-GFP, MoMkk1-RFP, and MoSep1^BACK^-GFP were co-expressed in wild-type Guy11, respectively, and proteins were detected using anti-RFP and anti-GFP antibodies. Lysed hyphal proteins were allowed to bind to RFP beads at 4°C for 4 h and then analyzed by IB with appropriate antibodies.

Since hyphal mitosis appears to be linked to MoMkk1 and its phosphorylation, we searched for MoMkk1 interacting proteins using both the yeast two-hybrid (Y2H) and co-immunoprecipitation (co-IP) approaches. We have identified a wide variety of potential interacting proteins, including cell cycle-associated proteins, mitochondrion proteins, carboxypeptidase, helicase, energy metabolism-related proteins, protein kinases, and proteins with unknown functions (S1 Table). Interestingly, a homolog of the yeast Ste/Ste11/Cdc15 protein kinase known to be associated with MEN functions [23] was identified and named as MoSep1 (MGG_04100) (S1A Fig). We again verified the interaction by Y2H and GST pull-down assays (Fig 2B and 2C). To demonstrate the interaction *in vivo*, we co-expressed MoMkk1-RFP and MoSep1-GFP fusion constructs into Guy11. We failed to detect the whole protein of MoSep1 *in vivo* by different methods. However, segmented MoSep1 proteins (STK and BACK) were able to purify and interact with MoMkk1 *in vivo* (Fig 2A–2D). MoSep1^STK^ is localized diffusely throughout the cell, MoSep1^BACK^ is localized to the spindle pole body (SPB) (S2 Fig). These localization patterns are similar to yeast Cdc15 [29].

### MoSep1, MoDbf2, and MoMob1 are involved in vegetative growth, conidiation, and virulence of *M*. *oryzae*

**Fig 3:**
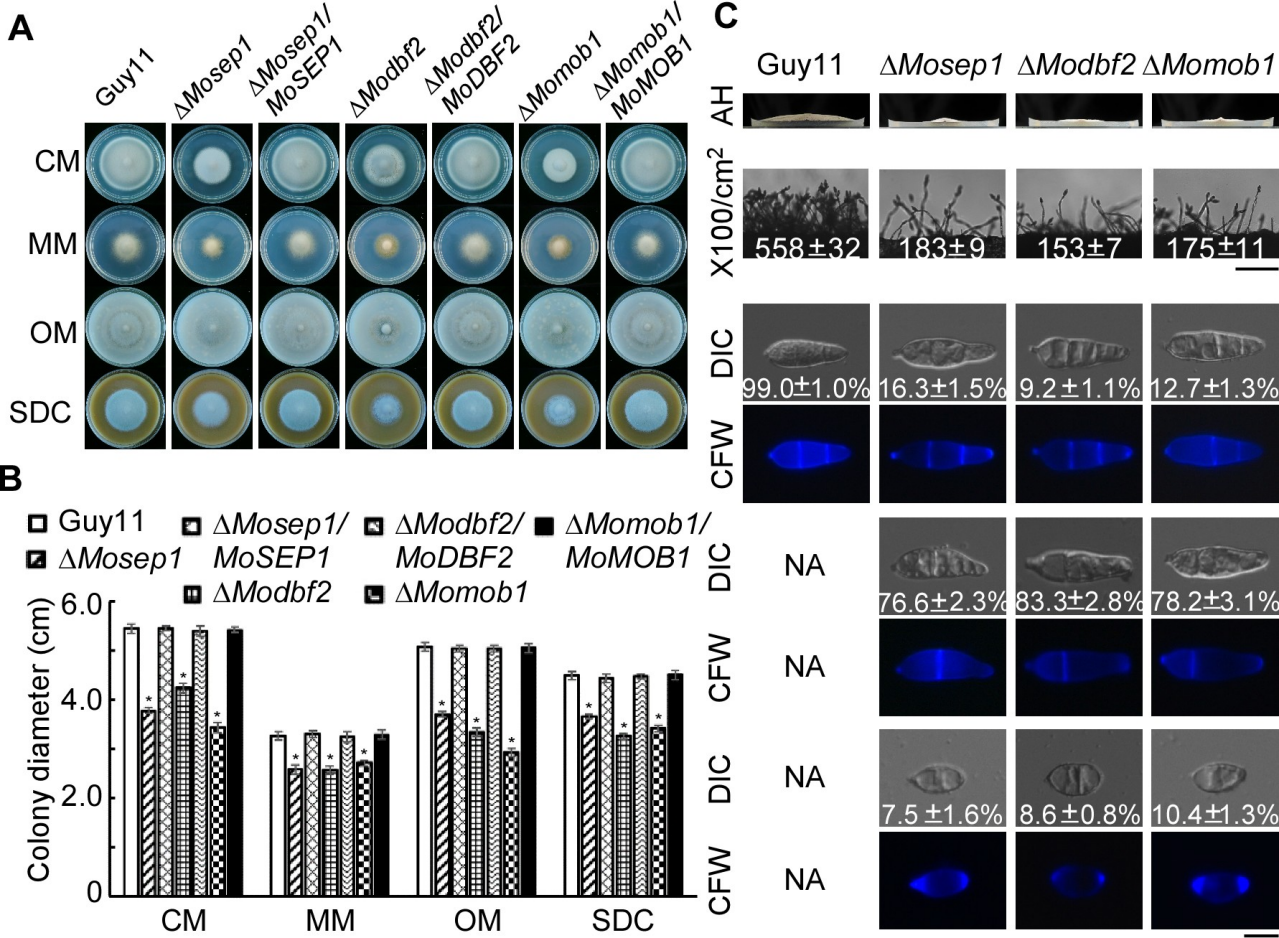
MEN components MoSep1, MoDbf2, and MoMob1 are involved in vegetative growth and conidiation of *M*. *oryzae*. (A) The Δ*Mosep1*, Δ*Modbf2*, and Δ*Momob1* mutants displayed significantly reduced mycelial growth on complete media (CM), minimal media (MM), oatmeal media (OM), and straw decoction and corn media (SDC) after incubation at 28°C for 7 days in darkness. (B) Statistical analysis of colony diameters from Guy11, Δ*Mosep1*, Δ*Modbf2*, and Δ*Momob1* mutants, and their corresponding complemented strains on different media. Error bars represent standard deviations from three independent experiments. Asterisks indicate statistical significance according to a Student’s test (p<0.01). (C) The first line shows aerial hyphae (AH) growth was reduced in the Δ*Mosep1*, Δ*Modbf2*, and Δ*Momob1* mutants after incubation for 10 days. The second line shows conidia formation under a light microscope 24 h at room temperature after induction of conidiation under coverslips. Bar, 50 μm. The bottom line shows conidia of Guy11, the Δ*Mosep1*, Δ*Modbf2*, and Δ*Momob1* mutants examined by differential interference contrast (DIC) and epifluorescence microscopy, respectively. NA, not available. Bar, 10 μm.

In yeast, the MEN kinase pathway consists of Cdc15 and Dbf2-Mob1 [33]. Since MoSep1 is the homolog of Cdc15, we extended the search and identified MoDbf2 (MGG_02757) and MoMob1 (MGG_03151) as the Dbf2 and Mob1 homologs of *M*. *oryzae* (S1B and S1C Fig). The mutant strains Δ*Modbf2*, Δ*Momob1*, and Δ*Mosep1* were respectively generated (S3A–S3D Fig). All mutants showed apparent attenuated growth after incubation at 28°C for 7 days in the dark (Fig 3–3C) and the defect in conidiation producing abnormal and approximately 33% conidia in comparison to Guy11 (Fig 3C). These results indicated that MEN plays a vital role in the growth and conidiation of the fungus.

**Fig 4:**
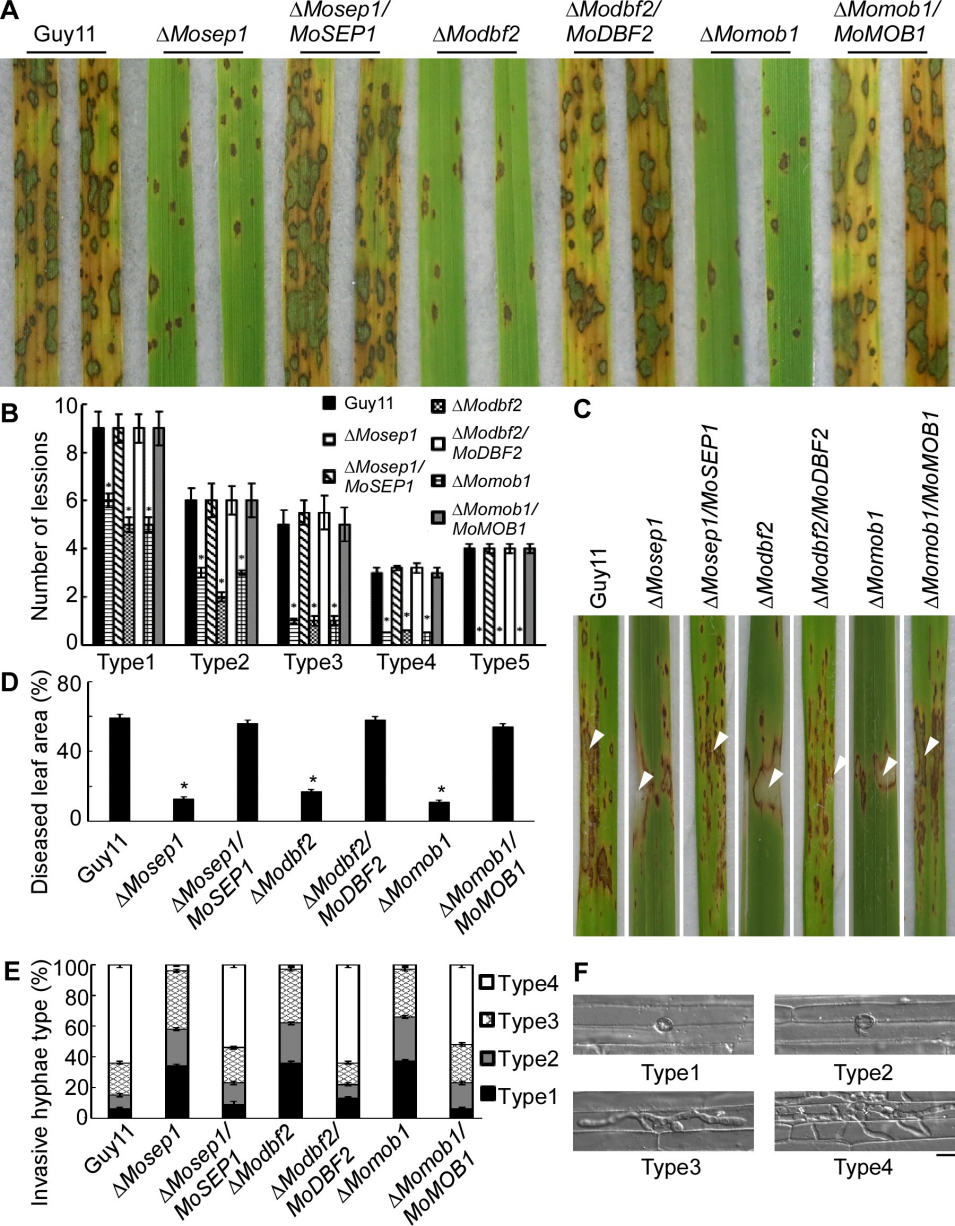
MoSep1, MoDbf2, and MoMob1 contribute to virulence. (A) Pathogenicity assay in rice. Two-week-old rice seedlings were inoculated conidial suspensions of Guy11, the Δ*Mosep1*, Δ*Modbf2*, Δ*Momob1* mutants, and their corresponding complemented strains and photographed at 5 days post-inoculation (dpi). (B) Quantification of lesion types (per 1.5 cm^2^) on susceptible rice spayed with conidia Guy11, the Δ*Mosep1*, Δ*Modbf2*, Δ*Momob1* mutants, and their corresponding complemented strains. Lesions were quantified by a ‘lesion-type’ scoring assay which divided the lesions into 1–5 types according to their severity (type 1, pinhead-sized dark brown specks without visible centers; type 2, small brown lesions that are approximately 1 mm in diameter; type 3, 2–3 mm gray spots with brown margins; type 4, elliptical gray spots longer than approximately 3–4 mm; type 5, large eyespot lesions that coalesced infecting 50% or more of the leaf area). Error bars represent the standard deviations from three independent experiments. Asterisks indicate statistical significance according to a Student’s test (p<0.01). (C) Pathogenicity was tested by rice injection assay with the conidia concentration of 5 × 10^4^ spores/ml and photographed at 7 dpi. The arrow points to the injection site. (D) Diseased leaf area analysis of (C). The data were presented as a bar chart showing the percentage of lesion areas. Error bars represent the standard deviations from three independent experiments. Asterisks indicate statistical significance according to a Student’s test (p<0.01). (E, F) Statistical analysis of the infectious hyphal type (type 1, no penetration; type 2, with penetration peg; type 3, with a single invasive hypha; type 4, with extensive hyphal growth) on rice leaf sheaths. Rice leaf sheaths were inoculated with conidial suspensions and examined at 36 h post-inoculation (hpi). One hundred infectious hyphae were counted for each strain and the experiment was repeated three times. Error bars represent the standard deviations.

In addition, the Δ*Modbf2*, Δ*Momob1*, and Δ*Mosep1* mutant strains were sprayed onto 14 days old rice seedlings (cv. CO39), along with Guy11 and the respective complemented strains (Δ*Mosep1*/*MoSEP1*, Δ*Modbf2*/*MoDBF2*, Δ*Momob1*/*MoMOB1*). The mutants caused fewer and smaller lesions by producing more type 1–3 (70%) and few type 4–5 (5%) lesions in comparison to ~ 25% type 4–5 lesions in Guy11 (Fig 4A and 4B). Similar results were observed in assays using the rice sheath injection assay (Fig 4C and 4D). Moreover, an excised leaf sheath assay was performed to detect the mutants’ infectious hyphae extension ability [34]. At 48 hours post-inoculation (hpi), all mutants’ invasive hyphae were restricted to a single cell, in contrast to Guy 11 whose invasive hyphae were free extending (Fig 4E and 4F). These results suggested that MoSep1, MoDbf2, and MoMob1 are all important for the pathogenicity of *M*. *oryzae*.

We then tested the appressorial turgor pressure using the incipient cytorrhysis [35] to examine the cause of virulence attenuation. At 1 M glycerol, the appressorium collapse rate was ~37% in the Δ*Mosep1*, Δ*Modbf2*, and Δ*Momob1* mutants compared with 11% in Guy11. With the increase of the external glycerol concentration, the proportion of collapsed appressoria remained higher in the mutants than in Guy11, indicating that mutant appressoria were defective in maintaining turgor pressure (S4A Fig). Since the turgor pressure is largely dependent on the breakdown of glycogen and the transfer of triacylglycerols [36], we estimated the cellular distribution of glycogen and lipid bodied using I_2_ and KI staining [36]. We found that glycogen breakdown was delayed by 10 h in the three mutants, compared to 6 h in Guy11 (S4B Fig). We also performed a Nile red staining assay to examine lipid body distribution [37] and found that lipid degradation was significantly postponed in the mutants than in Guy11. Degradation slowed down with numerous lipid bodies remaining in more than 55% of mature appressoria 24 h following germination in the mutants. In comparison, lipid body transfer into appressoria was completed 8 h after germination in >90% of appressoria by Guy11 (S4C Fig).

### MoSep1, MoDbf2, and MoMob1 are involved in MEN

**Fig 5:**
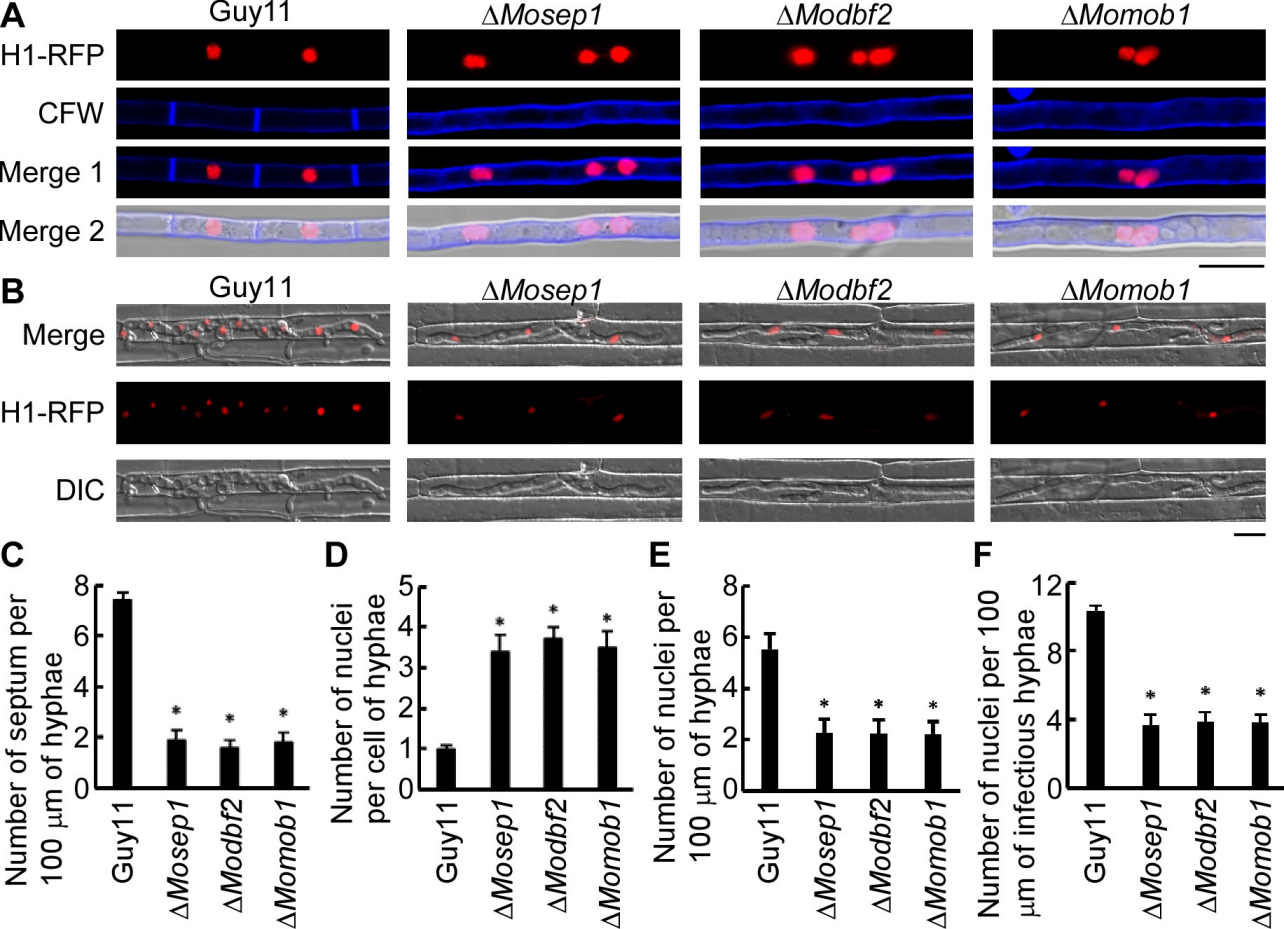
MoSep1, MoDbf2, and MoMob1 are involved in the Mitosis Exit Network (MEN). (A) Hyphae of Guy11 and the Δ*Mosep1*, Δ*Modbf2*, Δ*Momob1* mutants expressing the H1-RFP construct were stained with CFW and examined by epifluorescence microscopy. Bar, 10 μm. (B) Infectious hyphae of strains mentioned in (A) examined by epifluorescence microscopy. Bar, 10 μm. (C) Statistical analysis of the number of septa per 100 μm of hyphae in (A). One hundred hyphae were counted for each strain and the experiment was repeated three times. Error bars represent the standard deviations. Asterisks indicate statistical significance according to a Student’s test (p<0.01). (D) Statistical analysis of the number of nuclei per cell of hyphae in (A). One hundred hyphae were counted for each strain and the experiment was repeated three times. Error bars represent the standard deviations. Asterisks denote statistical significance according to a Student’s test (p<0.01). (E) Statistical analysis of the number of nuclei per 100 μm of hyphae in (A). One hundred hyphae were counted for each strain and the experiment was repeated three times. Error bars represent the standard deviations. Asterisks indicate statistical significance according to a Student’s test (p<0.01). (F) Statistical analysis of the number of nuclei per 100 μm of infectious hyphae of (B). One hundred infectious hyphae were counted for each strain and the experiment was repeated three times. Error bars represent the standard deviations. Asterisks indicate statistical significance according to a Student’s test (p<0.01).

To test the conserved roles of these three proteins in MEN of *M*. *oryzae* according to Saunders et al. [23], we introduced the histone H1-RFP gene fusion construct into these three mutants, respectively. Following CFW staining, we found that the hyphae have fewer and unevenly distributed septa (Fig 5A and 5C). They often contain multiple nuclei in each hyphal compartment and the number of nuclei per 100 μm hyphae was about 2–3 in the mutants compared to 4–6 nuclei in Guy11 (Fig 5A, 5D and 5E). These results indicated that these three mutants are defective in mitotic exit and the nuclei do not stop to divide.

Our previous results showed that all three mutants produce fewer and abnormal conidia. More than 76% conidia of these three mutants were two-celled instead of typical three-celled, and more than 75% had only two nuclei (Fig 3C and S5 Fig). Only one septum was observed in nearly all of the mutant conidia, in contrast to Guy11 whose conidia contain three nuclei with one nucleus per compartment. These findings suggested that MEN play significant functions in mitosis and cytokinesis of the conidia. To further explore this function, we assayed the appressorium formation ability on hydrophobic surfaces [38]. In Guy11, one nucleus moves back into the conidium following a single round of mitosis, and the other nucleus moves into the developing appressorium 6 h following germination. However, in the mutants, appressoria remain unformed, and the nuclei remained in the germinal tubes (S5 Fig). Infectious hyphae growth in rice sheath assays showed that the number of nuclei per 100 μm infectious hyphae was about 3–4 in the mutants compared to 8–11 nuclei in Guy11 (Fig 5B and 5F). All these results suggested that MoSep1, MoDbf2, and MoMob1 are required for mitotic exit and proper cytokinesis.

### Disruption of MEN results in defects in CWI

**Fig 6:**
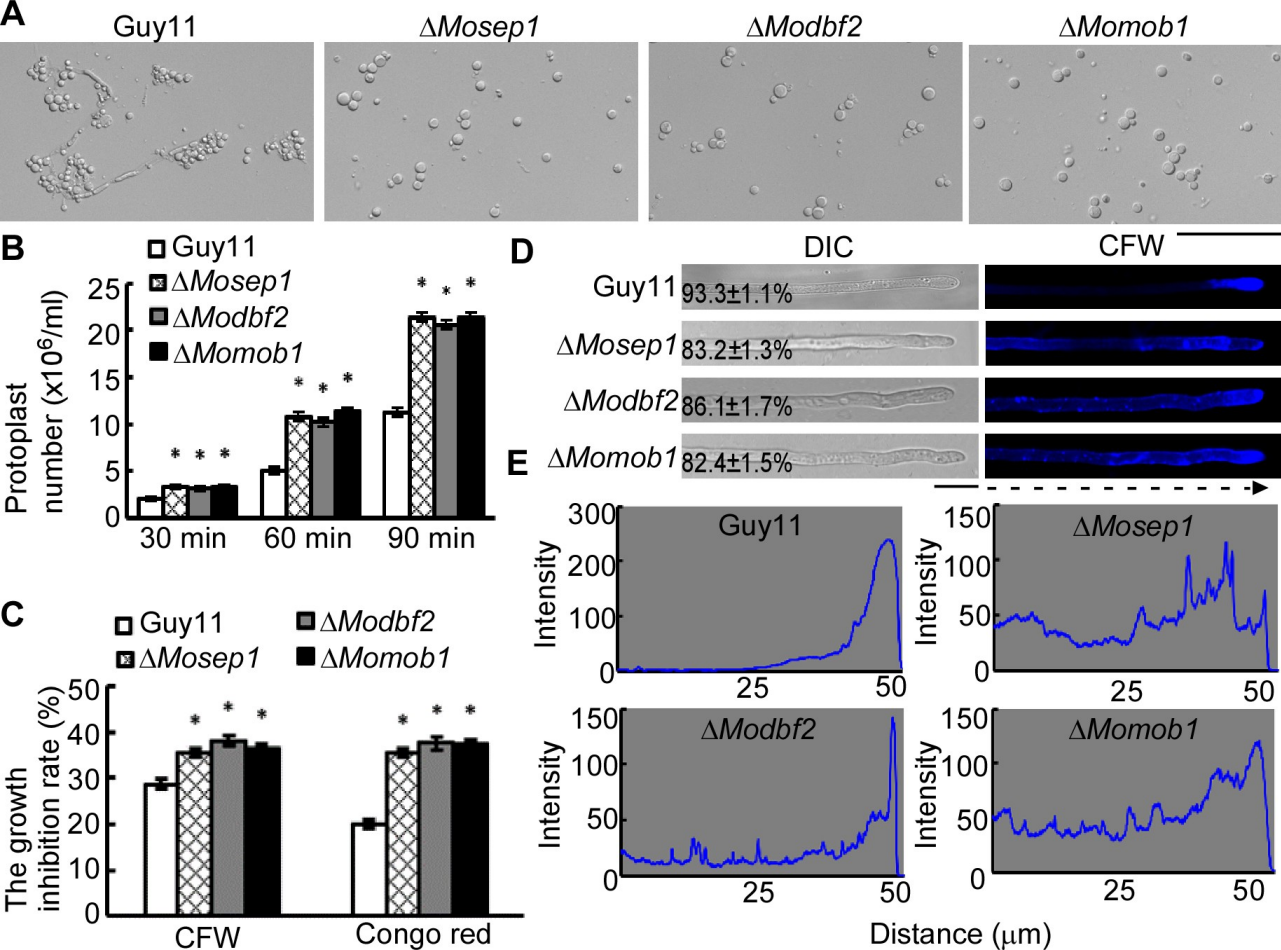
Disruption of MEN results in defects in CWI signaling. (A) Light microscopic examination of protoplast release of Guy11, Δ*Mosep1*, Δ*Modbf2*, and Δ*Momob1* mutants after treatment with cell wall-degrading enzymes for 45 min at 30°C. Bar, 50 μm. (B) Statistical analysis of protoplast release of Guy11, and the Δ*Mosep1*, Δ*Modbf2*, Δ*Momob1* mutants after treatment with cell wall-degrading enzymes for 30 min, 60 min, and 90 min at 30°C. Error bars represent the standard deviations from three independent experiments. Asterisks indicate statistical significance according to a Student’s test (p<0.01). (C) The hyphal diameter of Guy11, Δ*Mosep1*, Δ*Modbf2*, and Δ*Momob1* mutants examined 7 days after incubation on CM agar plates with different cell wall-perturbing agents including 400 μg/ml for CFW, and 400 μg/ml for Congo red. The experiments were repeated three times. Error bars represent the standard deviations from three independent experiments. Asterisks indicate statistical significance according to a Student’s test (p<0.01). (D) Observation of chitin in the cell wall. Hyphae of Guy11, Δ*Mosep1*, Δ*Modbf2*, and Δ*Momob1* mutants were stained with CFW for 5 min in darkness. Bar, 10 μm. CFW is widely used to stain cell wall chitin in fungi. (E) Linescan graph analysis of chitin stained with CFW of (D).

Since mitosis requires normal CWI activities including MoMkk1 phosphorylation [10], we wondered whether MoSep1, MoDbf2, or MoMob1 are involved in CWI signaling. We compared the impact of cell wall-degrading enzymes on mycelial cell walls and found that the hyphae of the Δ*Mosep1*, Δ*Modbf2*, and Δ*Momob1* mutants released more protoplasts than Guy11, suggesting more sensitive (Fig 6A and 6B). The mutant strains were also more susceptible to cell wall stress induced by CFW and Congo red (CR) (Fig 6C).

We used CFW staining [10,39] to test the cell wall properties of the three mutants and found that chitin distribution is uneven and not concentrated on the growing apices (Fig 6D and 6E). To detect whether MoSep1, MoDbf2, and MoMob1 are involved in chitin biosynthesis the regulation of chitin biosynthesis, we tested the expression levels of seven chitin synthase (CHS) genes and found six were significantly reduced (S6A Fig). We also estimated the chitin contents of Δ*Mosep1*, Δ*Modbf2*, and Δ*Momob1* mutants to be approximately 30% lower than that of Guy11 (S6B Fig). These results collectively suggested that MoSep1, MoDbf2, and MoMob1 are required for CWI functions and regulation.

### MoSep1 phosphorylates MoMkk1 and MoSep1-dependent MoMkk1 phosphorylation sites are vital for CWI

**Fig 7:**
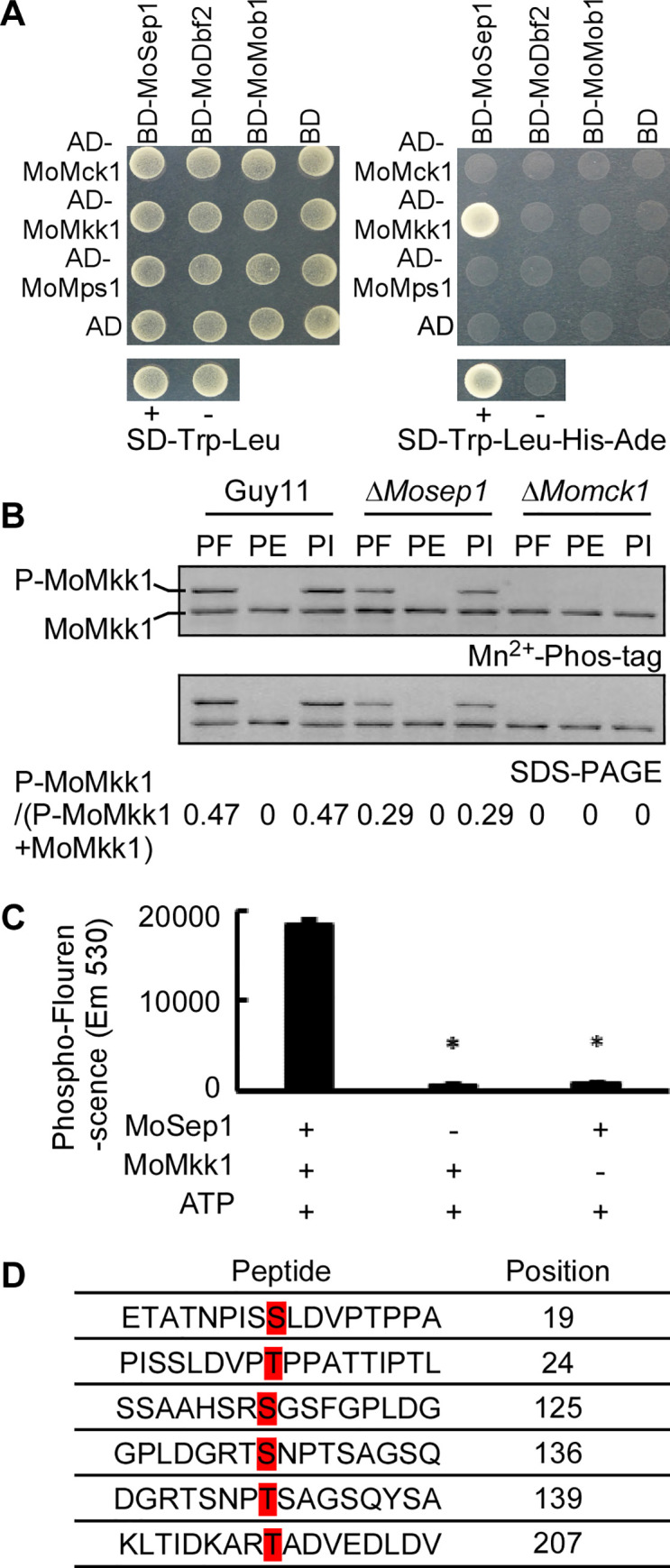
MoSep1 phosphorylates MoMkk1. (A) Yeast two-hybrid analysis between CWI components (*MoMCK1*, *MoMKK1*, and *MoMPS1*) with MEN components (*MoSEP1*, *MoDBF2*, and *MoMOB1*). pGADT7 and pGBKT7 fused with specific genes were co-introduced into yeast AH109 strain, and transformants were plated on SD-Leu-Trp as control and on SD-Leu-Trp-His-Ade for selection. (B) *In vivo* phosphorylation analysis of MoMkk1 in Guy11 and the Δ*Mosep1* mutant. MoMkk1-GFP proteins treated with phosphatase inhibitors (PI), phosphatase (PE), and detected by the anti-GFP antibody. MoMkk1 phosphorylation was estimated by calculating the amount of phosphorylated-MoMkk1 (P-MoMkk1) compared to total MoMkk1 (the numbers underneath the blot). (C) *In vitro* phosphorylation analysis by the fluorescence detection in tube (FDIT) method. Purified proteins of GST-MoMkk1, His-MoSep1 were used for protein kinase reaction in the presence of 50 μm ATP in a kinase reaction buffer and then dyed with Pro-Q Diamond Phosphorylation Gel Stain. Fluorescence signal at 590 nm (excited at 530 nm) was measured in a Cytation3 microplate reader (Biotek, Winooski, VT, USA). Error bars represent the standard deviations from three independent experiments. Asterisks indicate statistical significance according to a Student’s test (p<0.01). (D) MoMkk1 phosphorylation sites in the Guy11 compared with the Δ*Mosep1* mutant expressing *MoMKK1* was identified by LC-MS/MS analysis.

Given that the MEN pathway is linked to the CWI pathway and MoSep1 interacts with MoMkk1, we were curious about whether the MoSep1-MoMkk1 interaction mediates the link. We then tested but failed to establish any interactions of MoDbf2 and MoMob1 with MoMck1, MoMkk1, or MoMps1 through Y2H (Fig 7A). To further test whether MoSep1 phosphorylates MoMkk1 directly, Mn^2+^-Phos-tag SDS-PAGE was again performed. Results showed that the P-MoMkk1 band is weaker in the Δ*Mosep1* strain than in Guy11 but higher than the Δ*Momck1* mutant as MoMck1 normally phosphorylates MoMkk1 (Fig 7B). The further *in vitro* phosphorylation assay using protein gel-staining fluorescence dye supported that MoSep1 (His-MoSep1) could phosphorylate MoMkk1 (GST-MoMkk1) (Fig 7C).

We further determined the specific phosphorylation sites on MoMkk1 by MoSep1. MoMkk1-GFP proteins purified from the Δ*Mosep1* mutant and Guy11 were subject to mass spectrometry (MS) analysis that identified S19, T24, S125, S136, T139, T207 as the candidate phosphorylated sites (Fig 7D and S7 Fig). To validate this result, the phosphomimic mutation of MoMkk1^S19D, T24D, S125D, S136D, T139D, T207D^ (MoMkk1^6D^) was generated and transformed into the Δ*Mosep1* mutant. We found that MoMps1 phosphorylation level was partially restored (S8A Fig). Subsequently, the constitutively unphosphorylated MoMkk1^S19A, T24A, S125A, S136A, T139A, T207A^-GFP (MoMkk1^6A^-GFP for short) fusion constructs was generated and expressed in the Δ*Momkk1* mutant strain. The phosphorylation level of MoMps1 in the strain was partially reduced (S8B Fig). *In vitro* phosphorylation assays using purified GST-MoMkk1^S19A^, GST-MoMkk1^T24A^, GST-MoMkk1^S125A^, GST-MoMkk1^S136A^, GST-MoMkk1^T139A^, GST-MoMkk1^T207A^, GST-MoMkk1^6A^, GST-MoMkk1, and His-MoSep1 proteins all showed lowered phosphorylation levels (S8C Fig). All these results collectively suggested that these six sites are all important for the phosphorylation of MoMkk1 by MoSep1.

To further verify if this MoSep1-dependent phosphorylation is involved in regulating the CWI pathway, we again estimated protoplast release from these strains. Δ*Mosep1* hyphae released more protoplasts than Δ*Mosep1/MoMKK1*^*6D*^, and Δ*Momkk1/ MoMKK1*^*6A*^ hyphae released more protoplasts than Guy11 (S9A and S9B Fig). Consistently, Δ*Momkk1/MoMKK1*^*6A*^ was more sensitive to Congo red (S9C Fig), and its chitin distribution was uneven and not restricted to the growth apices than controls (S9D Fig). These results indicated that the phosphorylation of MoMkk1 by MoSep1 plays an essential role in the conserved function of MoMkk1 in CWI signaling.

### MoSep1-dependent MoMkk1 phosphorylation is important for vegetative growth, conidiation, and pathogenicity in *M*. *oryzae*

**Fig 8:**
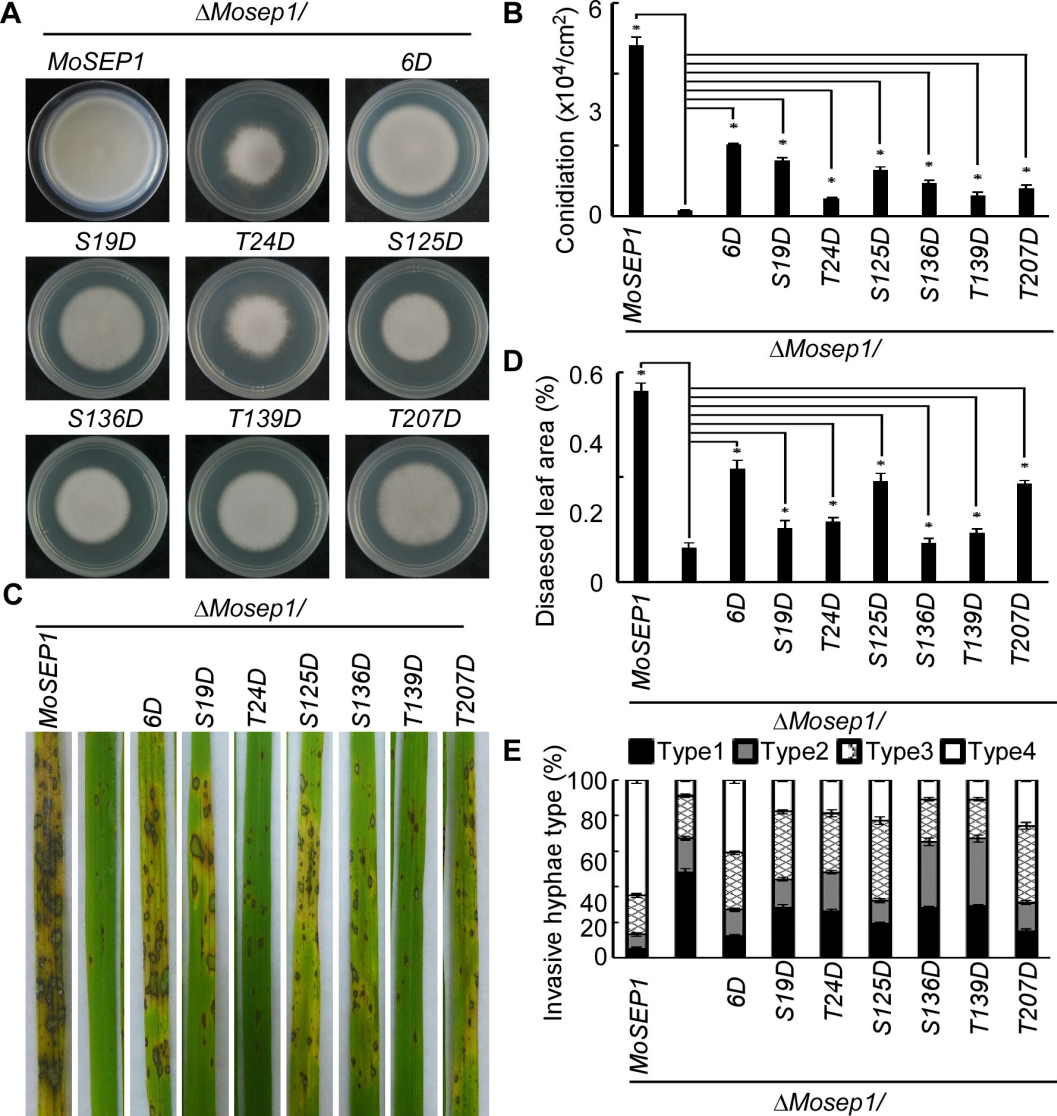
MoSep1-dependent MoMkk1 phosphorylation is vital for vegetative growth, conidiation, and pathogenicity in *M*. *oryzae*. (A) Growth of Δ*Mosep1/MoSEP1*, Δ*Mosep1*, Δ*Mosep1/MoMKK1*^*6D*^ (Δ*Mosep1/MoMKK1*^*S19D*, *T24D*, *S125D*, *S136D*, *T139D*, *T207D*^), Δ*Mosep1/MoMKK1*^*S19D*^, Δ*Mosep1/MoMKK1*^*T24D*^, Δ*Mosep1/MoMKK1*^*S125D*^, Δ*Mosep1/MoMKK1*^*S136D*^, Δ*Mosep1/MoMKK1*^*T139D*^, Δ*Mosep1/MoMKK1*^*T207D*^ mutant strains on CM medium. (B) Statistical analysis of conidia production on SDC medium cultured at 28°C for 7 days in the dark followed by 3 days of continuous illumination under fluorescent light. Error bars represent the standard deviations from three independent experiments. Asterisks indicate statistical significance according to a Student’s test (p<0.01). (C) Pathogenicity analysis using rice spraying assays and photographed at 7 dpi. (D) Diseased leaf area analysis of (C). Data were presented as a bar chart showing the percentage of lesion areas. Error bars represented the standard deviations from three independent experiments. Asterisks denote statistical significance according to a Student’s test (p<0.01). (E) Statistical analysis of the infectious hyphal type (type 1, no penetration; type 2, with penetration peg; type 3, with a single invasive hypha; type 4, with extensive hyphal growth) on rice leaf sheaths. Rice leaf sheaths were inoculated with conidial suspensions and examined at 36 h post-inoculation (hpi). One hundred infectious hyphae were counted for each strain and the experiment was repeated three times. Error bars represented the standard deviations.

As MoSep1-dependent MoMkk1 phosphorylation is involved in CWI signaling, we further examined its role in vegetative growth, conidiation, and virulence of the aforementioned phosphomimic mutants. Results showed that MoMkk1^S19D^-GFP, MoMkk1^S125D^-GFP, MoMkk1^S136D^-GFP, MoMkk1^T139D^-GFP, and MoMkk1^T207D^-GFP suppressed the growth defects of the Δ*Mosep1* mutant (Fig 8A). MoMkk1^S125D^-GFP and MoMkk1^T207D^-GFP also suppressed the conidiation defect (Fig 8B), and MoMkk1^S19D^-GFP, MoMkk1^S125D^-GFP, MoMkk1^S136D^-GFP, and MoMkk1^T207D^-GFP restored the defect in virulence (Fig 8C–8E). Although MoMkk1^T24D^-GFP failed to show suppression in growth (Fig 8A), it could restore the defect in conidiation and virulence partially (Fig 8B–8E). The constitutively activated MoMkk1^6D^-GFP showed the most suppression effect (Fig 8A–8E).

**Fig 9:**
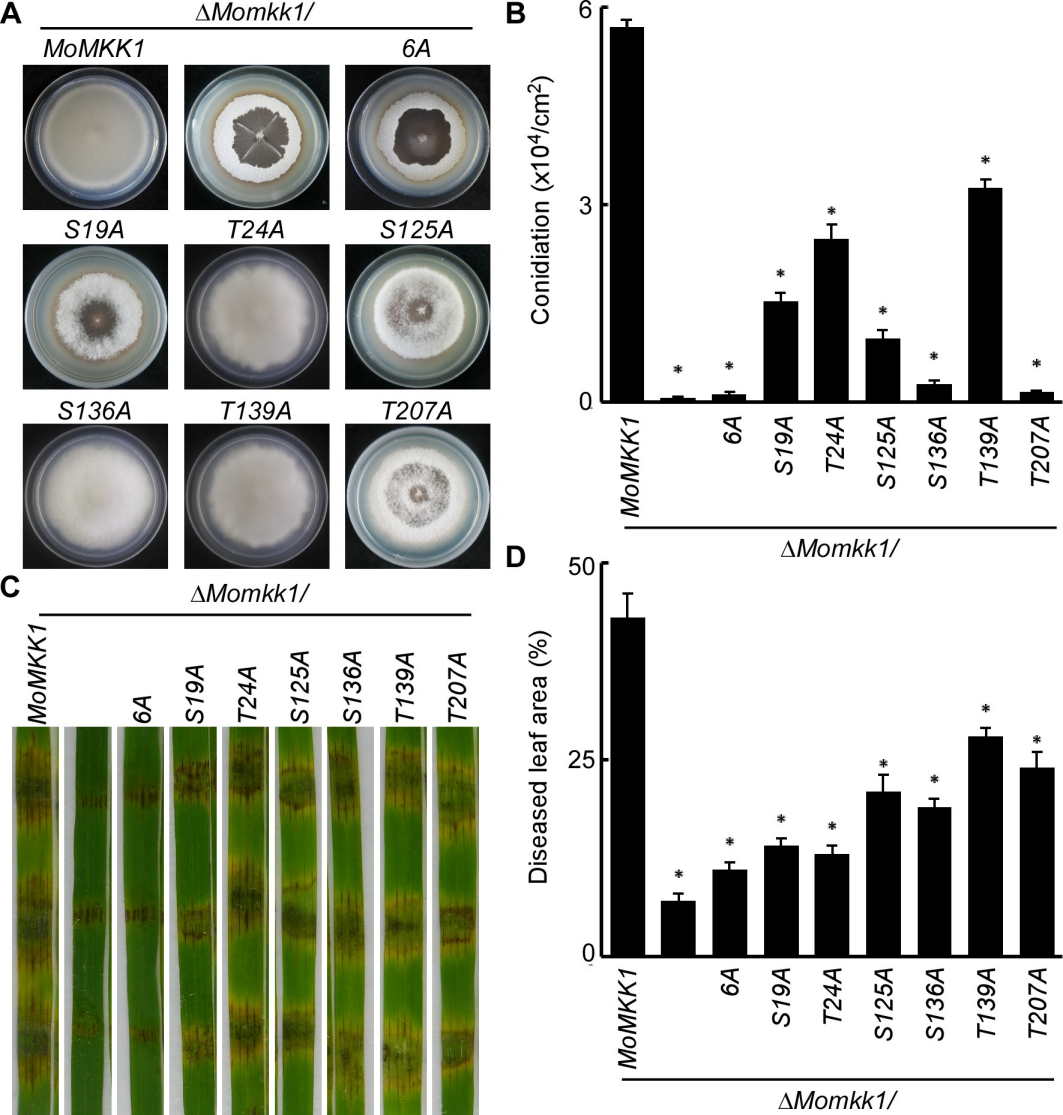
Disruption of MoSep1-dependent MoMkk1 phosphorylation leads to hyphal autolysis, reduced conidiation, and attenuated virulence. (A) Autolysis observation of Δ*Momkk1/MoMKK1*, Δ*Momkk1*, Δ*Momkk1/MoMKK1*^*6A*^ (Δ*Momkk1/MoMKK1*^*S19A*, *T24A*, *S125A*, *S136A*, *T139A*, *T207A*^), Δ*Momkk1/MoMKK1*^*S19A*^, Δ*Momkk1/MoMKK1*^*T24A*^, Δ*Momkk1/MoMKK1*^*S125A*^, Δ*Momkk1/MoMKK1*^*S136A*^, Δ*Momkk1/MoMKK1*^*T139A*^, Δ*Momkk1/MoMKK1*^*T207A*^ mutant strains on CM medium. Hyphae autolysis is a process of self-digestion of hyphal cultures. The part of the aerial hyphae with autolysis appeared watery collapse. (B) Statistical analysis of conidia production on SDC medium cultured at 28°C for 7 days in the dark followed by 3 days of continuous illumination under fluorescent light. Error bars represent the standard deviations from three independent experiments. Asterisks indicate statistical significance according to a Student’s test (p<0.01). (C) Pathogenicity test on rice leaves. Wounded rice leaves were incubated with different strains. Diseased leaves were photographed 4 days after inoculation. (D) Diseased leaf area analysis of (C). Data were presented as a bar chart showing the percentage of lesion areas. Error bars represent the standard deviations from three independent experiments. Asterisks indicate statistical significance according to a Student’s test (p<0.01).

We further tested the phenotypes of constitutively unphosphorylated strains. We found that the transformants of MoMkk1^S19A^-GFP, MoMkk1^S125A^-GFP, and MoMkk1^T207A^-GFP undergo progressive hyphal autolysis compared with the abundant aerial hyphae of the wild type, they are similar to the Δ*Momkk1* mutant (Fig 9A). The conidia of MoMkk1^S136A^-GFP and MoMkk1^T207A^-GFP were severely reduced, similar to the Δ*Momkk1* mutant (Fig 9B). Further pathogenicity tests using mycelial fragments to inoculate detached wounded rice leaves showed that the virulence of these mutants varies (Fig 9C and 9D), with inactivation of these six phosphorylation sites concurrently showed the most serious defects. The phosphomimic mutation of MoMkk1^6D^ can completely restore the defect in growth, conidiation, and virulence of Δ*Momkk1* mutant as a control (S10 Fig). These results indicated that the MoSep1-dependent MoMkk1 phosphorylation is essential for the development and pathogenicity of *M*. *oryzae*.

### MoSep1-dependent MoMkk1 phosphorylation is important for MEN in *M*. *oryzae*

**Fig 10:**
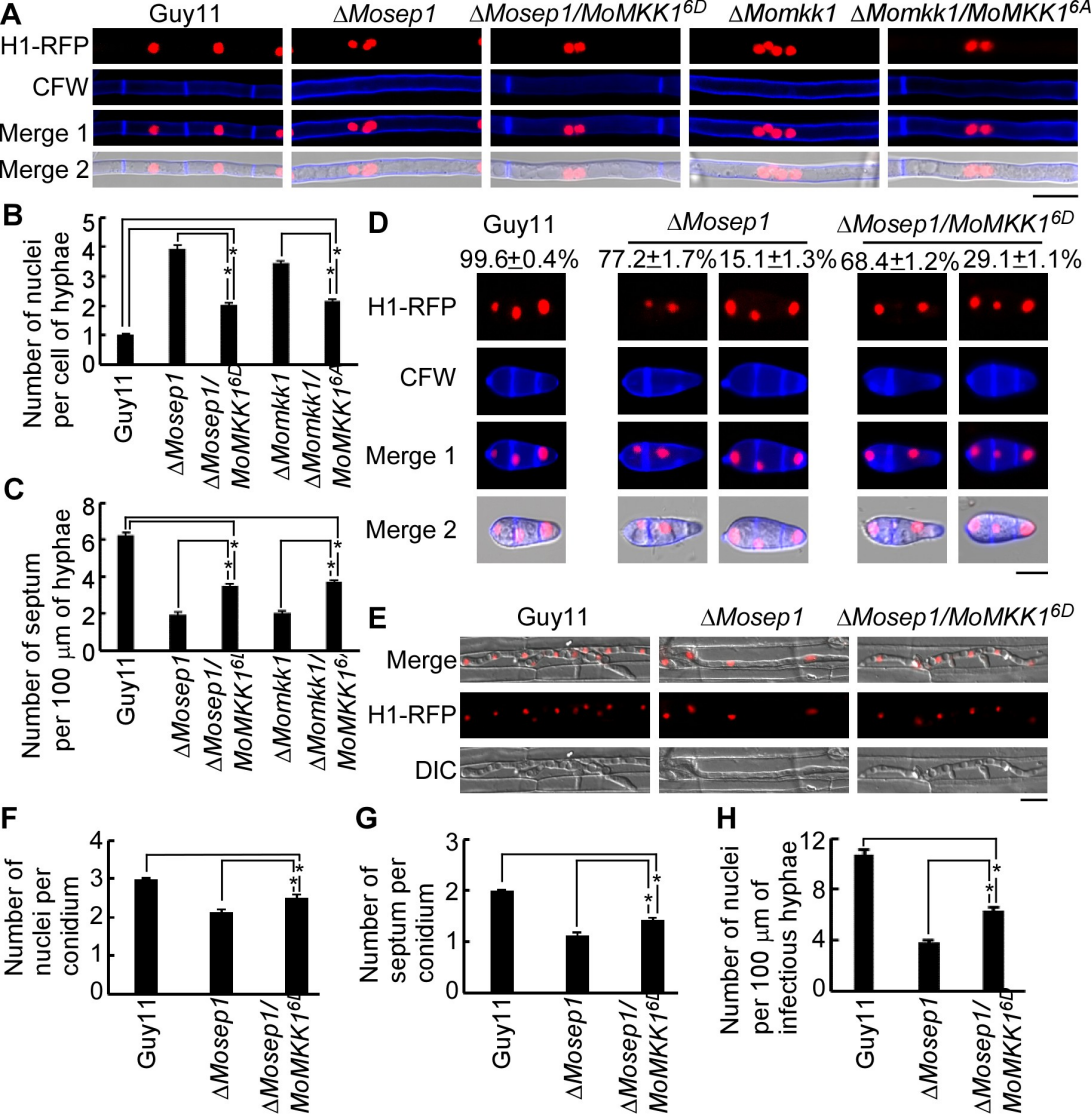
MoSep1-dependent MoMkk1 phosphorylation is vital for MEN in *M*. *oryzae*. (A) Hyphae of transformants of Guy11, the Δ*Mosep1*, Δ*Mosep1/MoMKK1*^*6D*^, Δ*Momkk1*, Δ*Momkk1/MoMKK1*^*6A*^ mutants expressing the H1-RFP construct were stained with CFW and examined by epifluorescence microscopy. Bar, 10 μm. (B) Statistical analysis of the number of nuclei per cell of hyphae in (A). One hundred hyphae were counted for each strain and the experiment was repeated three times. Error bars represent the standard deviations. Asterisks indicate statistical significance according to a Student’s test (p<0.01). (C) Statistical analysis of the number of septa per 100 μm of hyphae in (A). One hundred hyphae were counted for each strain and the experiment was repeated three times. Error bars represent the standard deviations. Asterisks indicate statistical significance according to a Student’s test (p<0.01). (D) Conidia of transformants of Guy11, the Δ*Mosep1*, Δ*Mosep1/MoMKK1*^*6D*^ mutants expressing the H1-RFP construct were stained with CFW and examined by epifluorescence microscopy. The values mean proportion of corresponding type conidia in the total for each strain. Bar, 10 μm. (E) Infectious hyphae of Guy11, the Δ*Mosep1*, Δ*Mosep1/MoMKK1*^*6D*^ mutants expressing the H1-RFP construct examined by epifluorescence microscopy. Bar, 10 μm. (F) Statistical analysis of the number of nuclei per conidium in (D). One hundred conidia were counted for each strain and the experiment was repeated three times. Error bars represent the standard deviations. Asterisks indicate statistical significance according to a Student’s test (p<0.01). (G) Statistical analysis of the number of septum per conidium in (D). One hundred conidia were counted for each strain and the experiment was repeated three times. Error bars represent the standard deviations. Asterisks indicate statistical significance according to a Student’s test (p<0.01). (H) Statistical analysis of the number of nuclei per 100μm infectious hypha in (E). One hundred infectious hyphae were counted for each strain and the experiment was repeated three times. Error bars represented the standard deviations. Asterisks indicate statistical significance (p<0.01).

As the constitutive activation of MoMkk1 phosphorylated sites suppresses the defects of the Δ*Mosep1* mutant, we were interested in testing whether such phosphorylation sites are also involved in the regulation of the MEN pathway. The H1-RFP construct was transformed into the Δ*Mosep1*/*MoMKK1*^*6D*^ and Δ*Momkk1/MoMKK1*^*6A*^ strains. When stained with CFW, additional septa were observed in the Δ*Mosep1/MoMKK1*^*6D*^ strain than in Δ*Mosep1* mutant and fewer septa in the Δ*Momkk1/MoMKK1*^*6A*^ strain than Guy11 (Fig 10A–10C), suggesting that the activation of the MoSep1-dependent phosphorylation sites is important for the formation of septa. In addition, more than one nucleus per hyphae cell was observed in the Δ*Momkk1/MoMKK1*^*6A*^ strain in comparison to one nucleus per cell of the normal hyphae (Fig 10A–10C) shows that the inactivated mutations MoMkk1^6A^ cannot restore the detect of Δ*Momkk1*. The abnormal conidia of the Δ*Mosep1/MoMKK1*^*6D*^ strain were fewer than those in the Δ*Mosep1* mutant (Fig 10D, 10F and 10G). The infectious hyphae extension ability was more robust in the Δ*Mosep1/MoMKK1*^*6D*^ strain than in the Δ*Mosep1* mutant, and 6–7 nuclei per 100 μm of infectious hyphae were more similar to the wild type strain (Fig 10E and 10H). These results further indicated the importance of MoSep1-dependent MoMkk1 activation in the cell cycle and mitosis of *M*. *oryzae*.

## Discussion

**Fig 11:**
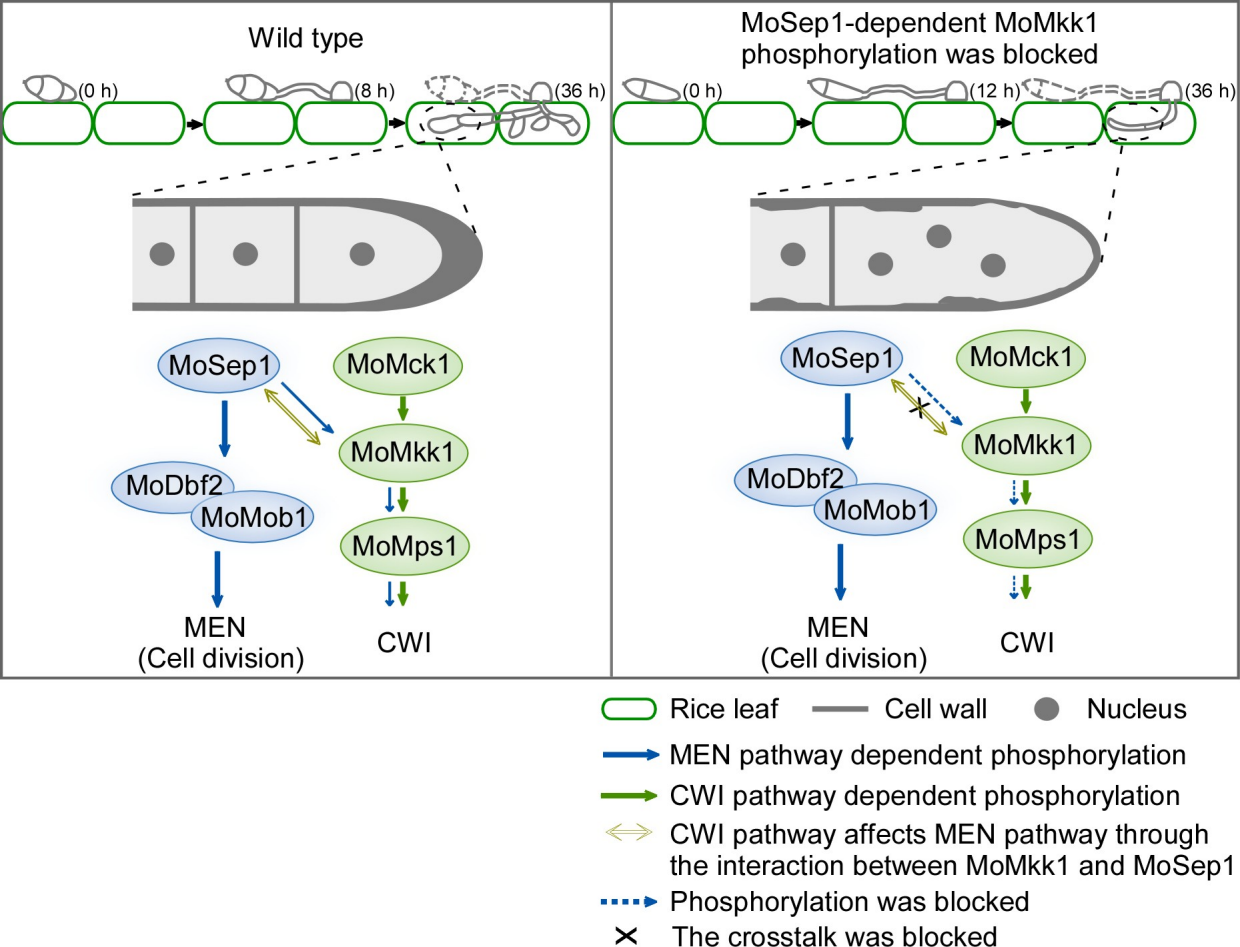
*M*. *oryzae* ensures cell wall integrity and normal progress of cell division through MoSep1-mediated MoMkk1 phosphorylation. Germination of conidia and expansion of infectious hyphae are accompanied by cell division and the cell wall synthesis. Normally, the MEN pathway cross talks with the CWI pathway through MoSep1 specific phosphorylation of MoMkk1, completing cell division and concurrently ensuring cell wall integrity during infection. However, the crosstalk between cell division and CWI cannot respond when the interaction of MoSep1 and MoMkk1 is blocked. Septa could not be normally formed, multiple nuclei per cell, and disordered distribution of cell wall chitin led to disrupted cell division and impaired CWI. The abnormality results in loss or attenuation of pathogenicity.

The cell wall is essential for maintaining cell morphology and functioning as a barrier against extracellular stress [10,40]. Balancing the rigidity or integrity while allowing growth-related expansion or plasticity remains the most important and sought after research projects [41–44]. This study found that the cell cycle-related kinase MoSep1 phosphorylates the cell wall integrity (CWI) pathway component MoMkk1 to link the mitotic exit network (MEN) with CWI signaling. This connection provides a means to balance the rigidity with the cell wall’s remodeling to allow cell division and growth. Significantly, MoSep1-MoMkk1 mediates the MEN and CWI crosstalk necessary for the virulence of *M*. *oryzae* (Fig 11). Furthermore, we found that the deletion of *MoDBF2* and *MoMOB1* that are downstream of MoSep1 also resulted in CWI defects. We speculated that there might be other connections between the cell wall integrity and cell division. There is still much to explore between these two essential pathways.

The cell cycle is a fundamental biological process that underlies the growth and development [45] and the entry, maintenance, and exit are all tightly regulated processes [46–47].Cdc15, a MoSep1 homolog, a vital kinase of the mitosis exit network that integrate Tem1 G-protein cycle and Cdc5 synthesis/degradation cycle to ensure mitosis exit after normal genome division [29]. Meanwhile, CWI is the key pathway regulating the adaptive response to cell wall biogenesis, morphogenesis, and stress. During cell elongation, the CWI detection system will adjust to the cell wall’s stress, providing the possibility that the maintenance of cell wall integrity is associated with the cell cycle [30,48]. Many previous studies have found contacts between cell wall synthesis and the cell cycle. For example, the plant cell wall thickness could influence polarity growth during cell division [31]. Newer technologies, such as a sub-resolution method to monitor cell wall thickness dynamics in living cells, were also used to detect cell wall dynamics during growth and division, revealing the connection between mitosis and cell wall remodeling [30]. Yeast cell wall damage promotes a checkpoint-like mechanism inhibiting DNA replication via Mck1-dependent Cdc6 degradation [49]. Here, we found that the deletion of MEN components also severely hindered cell wall function and decreased CWI activities (marked by MoMkk1 phosphorylation). This is the first time for a relationship between CWI and mitosis to be revealed in fungi. We further demonstrated that the link is established in a phosphorylation manner through the specific interaction between the MEN kinase MoSep1 and the CWI component MoMkk1. Specific MoSep1-dependent MoMkk1 phosphorylation sites were also identified and these roles in the development and virulence of *M*. *oryzae* revealed, proving that the crosstalk between MEN and CWI is important for the rice blast fungus.

During the growth and infection of *M*. *oryzae*, it is challenging how to relieve the stress originated from cell elongation. Studies have shown that some MEN components required for the G1/S phase progression are essential for maintaining proper appressorium development and pathogenicity of *M*. *oryzae* [22–23]. At the same time, the CWI pathway is also indispensable for the morphogenesis of aerial hyphae and fungal infection since the deletion of any CWI MAPK cascade components resulted in autolysis and attenuated virulence [10]. We found that it is difficult to detect the phosphorylation of MoMkk1 in the absence of MoMck1 (Fig 7B). Considering the importance of this conserved MAPK cascade, we wondered whether other kinases necessitate MoMck1 in order to phosphorylate MoMkk1 or that MoMkk1 phosphorylation is hard to detect *in vivo*. We have purified MoSep1 and MoMkk1 proteins *in vitro*, and found that MoSep1 can phosphorylate MoMkk1 without the presence of MoMck1. A previous study revealed that MoAtg1-mediated MoMkk1 phosphorylation could not be detected in the Δ*Momck1* mutant *in vivo*, but the detection improves when cells are under dithiothreitol (DTT)-induced stress. We reasoned that MoMkk1 phosphorylation by various kinases, including MoMck1, MoAtg1, or MoSep1, is an independent and specific event. Based on that these independent biological processes are respectively important, we further revealed that the link between the MEN and CWI pathways is equally vital. The MoSep1-MoMkk1 interaction ensures the proper response to the stress caused by cell elongation that underlies the fungal growth, development, and virulence.

We previously found that MoMkk1 also mediates crosstalk between the CWI pathway and other pathways, including the unfolded protein response (UPR) pathway, the high osmolality glycerol (HOG) MAPK pathway, and autophagy [10]. We demonstrated that the autophagy-related kinase MoAtg1 phosphorylates MoMkk1 under ER stress [16]. By identifying the cell cycle-related kinase MoSep1 that phosphorylates MoMkk1 to enhance the CWI activity, we revealed the complex and multitude of signaling pathway crosstalk surrounding CWI. The concurrent inactive phosphorylation sites mutation of MoSep1- and MoMck1-dependent mutant strains abolishing MoMps1 phosphorylation allowed us to conclude that MoSep1 and MoMck1 significantly contribute MoMkk1 phosphorylation under normal conditions. Such a conclusion implicates that the function of CWI in response to cell cycle-related stress plays an important part in the growth and pathogenicity of the blast fungus. Given such an important role, further investigation of MEN-CWI crosstalk is highly warranted.

## Material and methods

### Strains and culture conditions

The *M*. *oryzae* Guy11 strain was used as the wild type (WT) in this study. All strains were cultured on complete medium (CM) agar plates in the dark at 28°C, unless indicated otherwise [10]. Fungal mycelia were harvested from liquid CM for 1 day or 2 days at 28°C for microscopic observation, DNA, RNA, and protein extraction.

### Epifluorescence microscopy and nucleus staining and CFW staining

*M*. *oryzae* cells, including hyphae, conidia, and appressorium expressing fluorescent fusion proteins were incubated under appropriate conditions. The constructs, including H1-RFP, MoSep1^STK^-GFP, MoSep1^BACK^-GFP, and other phosphorylation mutations were transformed into Δ*Momck1*, Δ*Momkk1*, Δ*Momps1*, Δ*Mosep1*, Δ*Modbf2*, and Δ*Momob1* mutants or the wild type Guy11 strain. Epifluorescence microscopy was performed using a Zeiss LSM710 microscope. To visualize nuclei, DAPI was used at a concentration of 1 mg/ml in room temperature in darkness for 5 min. For CFW staining, mycelia were stained with 10 mg/ml for 5 min in the dark.

### Co-immunoprecipitation (Co-IP) assay

The MoMkk1-GFP fusion construct was transferred into Guy11. Total proteins were extracted and then incubated with anti-GFP beads. Proteins bounded to the beads were eluted and analyzed by MS. To confirm *in vivo* interactions involving MoMkk1-MoSep1^STK^, and MoMkk1-MoSep1^BACK^, the MoMkk1-RFP, MoSep1^STK^-GFP, and MoSep1^BACK^-GFP fusion constructs were constructed. Total proteins were isolated from transformants and incubated with anti-RFP beads. Proteins bounds to the beads were eluted and analyzed by Western blot with anti-GFP and anti-RFP antibodies.

### Yeast-two-hybrid (Y2H) assay

Full-length cDNA *MoMCK1*, *MoMKK1*, and *MoMPS1* was cloned into pGADT7 and cDNA of *MoSEP1* (*MoSEP1*^*STK*^ and *MoSEP1*^*BACK*^), *MoDBF2*, and *MoMOB1* genes was cloned into pGBKT7, respectively. The resulting constructs were first confirmed by DNA sequencing and then transformed in pairs into the yeast strain AH109 as previously described [16].

### GST-pull down assay

GST-MoMkk1, His, His-MoSep1, His-MoSep1^STK^, His-MoSep1^BACK^ were expressed in *Escherichia coli* BL21-CodonPlus (DE3) cells. Cells were lysed in lysis buffer (50 mM Tris, pH 8.0, 50 mM NaCl, 1mM PMSF) with a sonicator. Samples were centrifuged (14,000 rpm, 10 min) and the supernatants were transferred to a new 2 ml tube. The GST-MoMkk1 supernatants were then mixed with 100 μl glutathione Sepharose beads incubated at 4°C for 2 h. The recombinant GST-MoMkk1 bound to glutathione Sepharose beads was incubated with *E*. *coli* cell lysate containing His, His-MoSep1. His- MoSep1^STK^, His-MoSep1^BACK^ at 4°C for another 4 h. Finally, the beads were washed with buffer (50 mM Tris, pH 8.0, 50 mM NaCl, 1 mM PMSF, 1% Triton X-100) 5 times and eluted from the beads. Eluted proteins were analyzed by immunoblot (IB) with anti-His and anti-GST antibodies [50].

### Phosphorylation analysis

The MoMkk1-GFP fusion construct was transferred into Guy11 and Δ*Mosep1* mutant. The total protein extracted from mycelium was resolved on 8% SDS-PAGE prepared with 50 μm acrylamide-dependent Phos-tag ligand and 100 μm MnCl_2_ as described [16]. Gel electrophoresis was performed with a constant voltage of 80 V for 6–8 h. Gels were soaked in transfer buffer with 5–10 mM EDTA for 15 min three times before transferring, and followed by transfer buffer without EDTA for another 10 min two times. Protein transfer from the Mn^2+^-phos-tag acrylamide gel to the PVDF membrane was performed for ~48 h (depend on different proteins) at 80 V at 4°C, and then the membrane was analyzed by Western blotting using the anti-GFP antibody.

### Gene deletion and complementation

The gene deletion mutants were generated through the standard one-step gene replacement strategy [51]. To generate the *MoSEP1* gene replacement construct, 0.9-kb upstream and 1.1-kb downstream flanking sequences were amplified from *M*. *oryzae* genomic DNA. The resulting PCR products were digested with restriction endonucleases and ligated with the hygromycin resistance cassette (*HPH*) released from pCX62. The flanking sequences and the *HPH* cassette was amplified and transformed into Guy11 protoplasts. Putative mutants were verified by PCR. The complement fragment, which contains the entire *MoSEP1* gene coding region and its native promoter region, was amplified by PCR with primers and inserted into pYF11 to complement the mutant strain. The *MoDBF2* and *MoMOB1* gene deletion mutants were obtained using a similar strategy.

### Mass spectrometric analysis

To identify MoMkk1 interacting proteins. Total proteins were extracted from Guy11/*MoMKK1-GFP* transformants. Approximately 60 μl of anti-GFP beads was added into 2 ml diluted total protein samples to capture MoMkk1 interacting proteins. After incubated at 4°C overnight, the beads were washed 3 times with 700 μl PBS and proteins were eluted with 90 μl elution buffer (0.2 M glycine, pH 2.5). The eluent was immediately neutralized with 10 μl neutralization buffer (1.5 M Tris, pH 9.0). The eluted protein samples were subject to mass spectrometry analysis (Beijing Protein Innovation).

To identify phosphorylation sites of targeted proteins, total proteins were extracted from Guy11/*MoMKK1-GFP* and Δ*Mosep1/MoMKK1-GFP* transformants. Approximately 30 μl of anti-GFP beads was added into 1 ml diluted total protein samples. Following similar purification steps as above, the eluted proteins were neutralized and separated on 10% SDS-PAGE gel. The gel bands corresponding to the targeted proteins were excised from the gel and subject to mass spectrometry analysis as above.

### Plant infection assays

Conidia were adjusted to 5 × 10^4^ spores/ml in a 0.2% (w/v) gelation solution and 5 ml was sprayed on 2-week-old rice seedlings (*Oryza stative* cv. CO39). Inoculated plants were kept in a chamber at 25°C under 90% humidity for the first 24 h and followed by a light/dark cycle for 4–7 days. For the injection assay, conidia (5 × 10^4^ spores/ml) were injected into the 4-week-old rice sheath using a 1ml syringe. After 30~48 h incubation at 25°C, the infected sheath inner epidermis of were torn for observation. For those mutants that could not form conidia, mycelia cultured in liquid CM for 2 days were harvested and fragmented prior to inoculation inoculated on wounded rice leaves and kept in the same conditions as described above.

### Protoplast release assay

Mycelia cultured in liquid CM were filtered and collected with a filter cloth. They were then washed and resuspended in a liquid containing 10 mg/ml lysing enzyme. Protoplast release was observed in 0.5 h, 1 h, and 1.5 h with a light microscope. This experiment was repeated three times.

### Chitin content

Mycelial samples were freeze-dried, and 5 mg of the freeze-dried mycelia were treated in 1 ml 6% KOH at 80°C for 1.5 h. The treated samples were centrifuged (14,000 g, 10 min) and the precipitates were washed 3 times with PBS. Precipitates were then suspended in 0.5 ml of Mcllvaine’s buffer (pH 6). An aliquot of 100 μl (13 units) of *Streptomyces plicatus* chitinase was added, incubated at 37°C for 16 h, and then mixed with 100 μl of 0.27 M sodium borate (pH 9) before boiling for 10 min. 1 ml of 10 folds diluted Ehrlich’s reagent was then added and further incubated at 37°C for 20 min. A 1 ml sample was then transferred to a 2.5-ml plastic cuvette and absorbance at 585 nm was recorded.

### *In vitro* phosphorylation analysis

The His-MoSep1, GST-MoMkk1, GST-MoMkk1^S19A^, GST-MoMkk1^T24A^, GST-MoMkk1^S125A^, GST-MoMkk1^S136A^, GST-MoMkk1^T139A^, GST-MoMkk1^T207A^, GST-MoMkk1^6A^ were expressed in *Escherichia coli* BL21-CodonPlus (DE3) cells and purified. A rapid Fluorescence Detection in Tube (FDIT) method using Pro-Q Diamond Phosphorylation Gel Stain was used to analyze protein phosphorylation *in vitro*. First, 2 mg MoMkk1 (or the unphosphorylated mutations) was mixed with MoSep1 in a kinase reaction buffer (100 mM PBS, 1 mM ascorbic acid, pH 7.5, 10 mM MgCl_2_), with 50 mM ATP at 25°C for 1 h. 10 folds of cold acetone was then added to terminate the reaction. Casein was homogenized and suspended in Mili-Q water at a concentration of 0.2 mg/ml to stain the proteins. Briefly, 100 ml of Pro-Q Diamond was mixed with 10 ml of casein and the mixture was kept in dark 1 h. 10 folds of cold acetone was then added and the mixture was allowed to incubate overnight at -20°C. Proteins were precipitated by centrifugation at 14,000 rpm for 1 h at 4°C. Discard the supernatant. The pellet (proteins) was washed twice using 500 μl cold acetone. The pellet was dissolved in 200 μl of Mili-Q water and transferred to a 96 well plate. Fluorescence signal at 590 nm (exited at 530 nm) was measured using a Cytation3 microplate reader.

## Supporting information

S1 Table Identification of putative MoMkk1-interacting proteins. MoMkk1-GFP proteins were co-purified with anti-GFP beads and analyzed by (MS). The bait construct AD-MoMkk1 was used to screen a yeast two-hybrid cDNA library constructed with an RNA pool from various stages, including conidia and infectious hyphae (0, 2, 4, 8, 12 and 24 h). The table is the result of a combination of the two methods.
(XLSX)

S1 Fig Phylogenetic analysis of MoSep1, MoDbf2, MoMob1. (A) Phylogenetic analysis of MoSep1 from other organisms using CLUSTAL_W and the neighbor-joining tree was constructed by MEGA-X with 1000 bootstrap replicates. GenBank accession numbers and the corresponding species names are shown below: *N*. *crassa* (*Neurospora crassa* XP_961421.2), *N*.*tetrasperma* (*Neurospora tetrasperma* XP_009847293.1), *S*. *macrospora* (*Sordaria macrospora* XP_003348179.1), *C*. *ligniaria* (*Coniochaeta ligniaria* OIW23703.1), *M*. *oryzae* (*Magnaporthe oryzae* XP_003719734.1), *F*. *oxysporum* (*Fusarium oxysporum* XP_031039140.1), *V*. *alfalfae* (*Verticillium alfalfa* XP_003008948.1), *C*. *fioriniae* (*Colletotrichum fioriniae* EXF84805.1), *C*. *graminicola* (*Colletotrichum graminicola* XP_008096339.1), *P*. *fici* (*Pestalotiopsis fici* XP_007840167.1), *S*. *cerevisiae* (*Saccharomyces cerevisiae* AJO95441.1), *Homo sapiens* (*Homo sapiens* NP_001358839.1), *Mus musculus* (*Mus musculus* NP_036076.2).(B) Phylogenetic analysis of MoDbf2 from other organisms using CLUSTAL_W and the neighbor-joining tree was constructed by MEGA-X with 1000 bootstrap replicates. GenBank accession numbers and the corresponding species names are shown below: *C*. *graminicola* (*Colletotrichum graminicola* XP_008090722.1), *C*. *fioriniae* (*Colletotrichum fioriniae* EXF86141.1), *V*. *alfalfae* (*Verticillium alfalfae* XP_003007554.1), *M*. *oryzae* (*Magnaporthe oryzae* XP_003720996.1), *F*. *oxysporum* (*Fusarium oxysporum* XP_018237083.1), *C*. *ligniaria* (*Coniochaeta ligniaria* OIW31153.1), *P*. *fici* (*Pestalotiopsis fici* XP_007837343.1), *S*. *macrospora* (*Sordaria macrospora* XP_003347031.1), *N*. *crassa* (*Neurospora crassa* XP_964888.1), *N*. *tetrasperma* (*Neurospora tetrasperma* EGZ78496.1), *S*. *cerevisiae* (*Saccharomyces cerevisiae* GFP66221.1), *Mus musculus* (*Mus musculus* XP_011244534.1), *Homo sapiens* (*Homo sapiens* XP_016865715.1). (C) Phylogenetic analysis of MoMob1 from other organisms using CLUSTAL_W and the neighbor-joining tree was constructed by MEGA-X with 1000 bootstrap replicates. GenBank accession numbers and the corresponding species names are shown below: *N*. *crassa* (*Neurospora crassa* Q9P601.2), *N*.*tetrasperma* (*Neurospora tetrasperma* XP_009852011.1), *S*. *macrospora* (*Sordaria macrospora* XP_003350675.1), *C*. *ligniaria* (*Coniochaeta ligniaria* OIW27326.1), *P*. *fici* (*Pestalotiopsis fici* XP_007835384.1), *M*. *oryzae* (*Magnaporthe oryzae* XP_003716843.1), *F*. *oxysporum* (*Fusarium oxysporum* XP_018233055.1), *V*. *alfalfae* (*Verticillium alfalfae* XP_003002586.1), *C*. *graminicola* (*Colletotrichum graminicola* XP_008096216.1), *C*. *fioriniae* (*Colletotrichum fioriniae* EXF80986.1), *S*. *cerevisiae* (*Saccharomyces cerevisiae* EGA61903.1), *Mus musculus* (*Mus musculus* NP_081011.1), *Homo sapiens* (*Homo sapiens* NP_060691.2).
(TIF)

S2 Fig MoSep1^STK^-GFP is localized diffusely throughout the cell and MoSep1^BACK^-GFP is localized to the spindle pole body (SPB). Hyphae of transformants expressing the MoSep1^STK^-GFP and MoSep1^BACK^-GFP constructs in Guy11 respectively were stained with DAPI and examined by epifluorescence microscopy. Bar, 10 μm.
(TIF)

S3 Fig PCR analysis of *MoSEP1*, *MoDBF2* and *MoMOB1* deletion mutants. (A) Strategy of knocking out target gene in *M*. *oryzae* genome. The targe region was replaced with a 1.4-kb fragment containing the hygromycin B-resistance cassette (*HPH*) to create the mutant. The lines below the arrows indicated the verified fragments of the mutant. (B) PCR was used to validate the deletion of the *MoSEP1* gene. When verified by fragment 1 (a 0.5-kb fragment within *MoSEP1*), a 0.5-kb band was observed in the wild type but not in the Δ*Mosep1* mutant. When verified by fragment 2 (About 1.2-kb upstream fragment of the *MoMOB1* gene plus 0.1-kb fragment of *HPH*), the Δ*Mosep1* mutant (but not the wild-type) exhibited a 1.3-kb band characteristic of the gene replacement event. (C) PCR was used to validate the deletion of the *MoDBF2* gene. When verified by fragment 1 (a 0.5-kb fragment within *MoDBF2*), a 0.5-kb band was observed in the wild type but not in the Δ*Modbf2* mutant. When verified by fragment 2 (About 1.2-kb upstream fragment of the *MoMOB1* gene plus 0.1-kb fragment of *HPH*), the Δ*Modbf2* mutant (but not the wild-type) exhibited a 1.3-kb band characteristic of the gene replacement event. (D) PCR was used to validate the deletion of the *MoMOB1* gene. When verified by fragment 1 (a 0.5-kb fragment within *MoMOB1*), a 0.5-kb band was observed in the wild type but not in the Δ*Momob1* mutant. When verified by fragment 2 (About 1.2-kb upstream fragment of the *MoMOB1* gene plus 0.1-kb fragment of *HPH*), the Δ*Momob1* mutant (but not the wild-type) exhibited a 1.3-kb band characteristic of the gene replacement event.
(TIF)

S4 Fig MoSep1, MoDbf2, and MoMob1 are important for turgor pressure, glycogen transference, and intracellular lipid degradation. (A) Conidia were incubated on the hydrophobic surface. The cytorrhysis assay using various concentrations of glycerol (1–4 M). For each glycerol concentration, at least 100 appressoria were observed and the number of collapsed appressoria was counted from three independent experiments. Error bars represented the standard deviations. Asterisks denote statistical significance according to a Student’s test (p<0.01). (B) Conidia were incubated on the hydrophobic surface. Samples were stained with I_2_/KI solution at different time points and yellowish-brown glycogen deposits became visible immediately. Bar, 50 μm. (C) Conidia were incubated on the hydrophobic surface. Samples were stained for the presence of lipid bodied by Nile red. Bar, 50 μm.
(TIF)

S5 Fig The deletion of *MoSEP1*, *MoDBF2*, and *MoMOB1*, respectively caused abnormal conidia germination, and deficiency in Mitotic Exit Network. Conidia were allowed to germinate on the hydrophobic surface. Conidia germination of transformants of Guy11 and the Δ*Mosep1*, Δ*Modbf2*, Δ*Momob1* mutants expressing the H1-RFP construct were stained with CFW at different time points and examined by epifluorescence microscopy. Bar, 50 μm.
(TIF)

S6 Fig The deletion of *MoSEP1*, *MoDBF2*, and *MoMOB1* respectively interfers with normal chitin synthesis. (A) Transcription analysis of seven chitin synthases in *M*. *oryzae* using quantitative reverse transcription-polymerase chain reaction (qRT-PCR). (B) N-Acetylglucosamine (GlcNAc) determination by the fluorimetric Morgan-Elson method showed significantly decreased chitin content in the Δ*Mosep1*, Δ*Modbf2*, Δ*Momob1* mutants. Data represented three independent experiments, each performed three times. Error bars represented the standard deviations. Asterisks denote statistical significance according to a Student’s test (p<0.01).
(TIF)

S7 Fig MoSep1-dependent MoMkk1 phosphorylation sites identified by MS analysis. MoMkk1 phosphorylation sites in the Guy11 compared with the Δ*Mosep1* mutant expressing *MoMKK1* was identified by MS analysis.
(TIF)

S8 Fig Mutations of MoSep1-dependent MoMkk1 phosphorylation sites affect phosphorylation levels of MoMps1 *in vivo* and MoMkk1 *in vitro*. (A)Detection of the MoMps1 phosphorylation levels in Δ*Mosep1/MoSEP1*, Δ*Mosep1*, Δ*Mosep1/MoMKK1*^*6D*^ (Δ*Mosep1/MoMKK1*^*S19D*, *T24D*, *S125D*, *S136D*, *T139D*, *T207D*^) mutant strains. The key kinase of the CWI pathway MoMps1 was detected by binding of the P-P44/42 antibody, and the total MoMps1 was detected by the P44/42 antibody as a control. The extent of MoMps1 phosphorylation was estimated by calculating the amount of phosphorylated-MoMps1 (P-MoMps1) compared to the amount of MoMps1 (the numbers underneath the blot). (B) Detection of the MoMps1 phosphorylation levels in Δ*Momkk1/MoMKK1*, Δ*Momkk1*, Δ*Momkk1/MoMKK1*^*6A*^ (Δ*Momkk1/MoMKK1*^*S19A*, *T24A*, *S125A*, *S136A*, *T139A*, *T207A*^) mutant strains. The degree of phosphorylation of momps1 is estimated in the same way as (A). (C) *In vitro* phosphorylation analysis of fluorescence detection in tube (FDIT) method. GST-MoMkk1, constitutively unphosphorylated GST-MoMkk1^S19A^, GST-MoMkk1^T24A^, GST-MoMkk1^S125A^, GST-MoMkk1^S136A^, GST-MoMkk1^T139A^, GST-MoMkk1^T207A^, GST-MoMkk1^6A^ (MoMkk1^S19A, T24A, S125A, S136A, T139A, T207A^), His-MoSep1 fusion protein were obtained for *in vitro* phosphorylation analysis. Error bars represented the standard deviations from three independent experiments. Asterisks denote statistical significance according to a Student’s test (p<0.01).
(TIF)

S9 Fig MoSep1-dependent MoMkk1 phosphorylation is vital for CWI signaling. (A) Light microscopic examination of protoplast release of Guy11, the Δ*Mosep1*, Δ*Mosep1/MoMKK1*^*6D*^, Δ*Momkk1*, Δ*Momkk1/MoMKK1*^*6A*^ mutants after treatment with cell wall-degrading enzymes for 45 min at 30°C. Bar, 50 μm (B) Statistical analysis of protoplast release of Guy11, the Δ*Mosep1*, Δ*Mosep1/MoMKK1*^*6D*^, Δ*Momkk1*, Δ*Momkk1/MoMKK1*^*6A*^ mutants after treatment with cell wall-degrading enzymes for 30 min, 60 min, and 90 min at 30°C. Error bars represented the standard deviations from three independent experiments. Asterisks denote statistical significance according to a Student’s test (p<0.01). (C) The hyphal diameter of Guy11, the Δ*Mosep1*, Δ*Mosep1/MoMKK1*^*6D*^, Δ*Momkk1*, Δ*Momkk1/MoMKK1*^*6A*^ mutants examined 7 days after incubation on CM agar plates with cell wall-perturbing agents; 400 μg/ml for CFW, and 400 μg/ml for Congo red. The experiments were repeated three times. Error bars represented the standard deviations. Asterisks denote statistical significance according to a Student’s test (p<0.01). (D) Hyphae of Guy11, the Δ*Mosep1*, Δ*Mosep1/MoMKK1*^*6D*^, Δ*Momkk1*, Δ*Momkk1/MoMKK1*^*6A*^ mutants were stained with CFW for 5 min in the darkness. The distribution of chitin in the cell wall was disrupted.
(TIF)

S10 Fig Phosphomimic mutation of MoMkk1^6D^ completely rescues the defect in growth, conidiation, and virulence of Δ*Momkk1* mutant. (A) Autolysis observation of Δ*Momkk1/MoMKK1*, Δ*Momkk1*, Δ*Momkk1/MoMKK1*^*6D*^ (Δ*Momkk1/MoMKK1*^*S19D*, *T24D*, *S125D*, *S136D*, *T139D*, *T207D*^), mutant strains on CM medium. (B) Statistical analysis of conidia production on SDC medium cultured at 28°C for 7 days in the dark followed by 3 days of continuous illumination under fluorescent light. Error bars represent the standard deviations from three independent experiments. Asterisks indicate statistical significance according to a Student’s test (p<0.01). (C) Pathogenicity test on rice leaves. Wounded rice leaves were incubated with different strains. Diseased leaves were photographed 4 days after inoculation. (D) Diseased leaf area analysis of (C). Data were present as a bar chart showing the percentage of lesion areas. Error bars represented the standard deviations from three independent experiments. Asterisks indicate statistical significance according to a Student’s test (p<0.01).
(TIF)

## References

[1] Levin DE. Regulation of cell wall biogenesis in *Saccharomyces cerevisiae*: the cell wall integrity signaling pathway. Genetics. 2011;189(4):1145–75. 10.1534/genetics.111.128264 22174182 PMC3241422

[2] Höfte H, Voxeur A. Plant cell walls. Curr Biol. 2017;27(17):R865–r70. 10.1016/j.cub.2017.05.025 28898654

[3] Naseer S, Lee Y, Lapierre C, Franke R, Nawrath C, Geldner N. Casparian strip diffusion barrier in *Arabidopsis* is made of a lignin polymer without suberin. Proc Natl Acad Sci U S A. 2012;109(25):10101–6. 10.1073/pnas.1205726109 22665765 PMC3382560

[4] Gow NAR, Latge JP, Munro CA. The fungal cell wall: structure, biosynthesis, and function. Microbiol Spectr. 2017;5(3). 10.1128/microbiolspec.funk-0035-2016 PMC11687499 28513415

[5] Kang X, Kirui A, Muszyński A, Widanage MCD, Chen A, Azadi P, et al Molecular architecture of fungal cell walls revealed by solid-state NMR. Nat Commun. 2018;9(1):2747 10.1038/s41467-018-05199-0 30013106 PMC6048167

[6] Hohmann S. Osmotic stress signaling and osmoadaptation in yeasts. Microbiol Mol Biol Rev. 2002;66(2):300–72. 10.1128/mmbr.66.2.300-372.2002 12040128 PMC120784

[7] Denness L, McKenna JF, Segonzac C, Wormit A, Madhou P, Bennett M, et al Cell wall damage-induced lignin biosynthesis is regulated by a reactive oxygen species- and jasmonic acid-dependent process in *Arabidopsis*. Plant Physiol. 2011;156(3):1364–74. 10.1104/pp.111.175737 21546454 PMC3135913

[8] Bacete L, Mélida H, Miedes E, Molina A. Plant cell wall-mediated immunity: cell wall changes trigger disease resistance responses. Plant J. 2018;93(4):614–36. 10.1111/tpj.13807 29266460

[9] Vaahtera L, Schulz J, Hamann T. Cell wall integrity maintenance during plant development and interaction with the environment. Nat Plants. 2019;5(9):924–32. 10.1038/s41477-019-0502-0 31506641

[10] Yin Z, Tang W, Wang J, Liu X, Yang L, Gao C, et al Phosphodiesterase MoPdeH targets MoMck1 of the conserved mitogen-activated protein (MAP) kinase signalling pathway to regulate cell wall integrity in rice blast fungus *Magnaporthe oryzae*. Mol Plant Pathol. 2016;17(5):654–68. 10.1111/mpp.12317 27193947 PMC6638318

[11] Irie K, Takase M, Lee KS, Levin DE, Araki H, Matsumoto K, et al MKK1 and MKK2, which encode *Saccharomyces cerevisiae* mitogen-activated protein kinase-kinase homologs, function in the pathway mediated by protein kinase C. Mol Cell Biol. 1993;13(5):3076–83. 10.1128/mcb.13.5.3076 8386320 PMC359700

[12] Martín H, Arroyo J, Sánchez M, Molina M, Nombela C. Activity of the yeast MAP kinase homologue Slt2 is critically required for cell integrity at 37 degrees C. Mol Gen Genet. 1993;241(1–2):177–84 10.1007/BF00280215 8232202

[13] Lee KS, Levin DE. Dominant mutations in a gene encoding a putative protein kinase (BCK1) bypass the requirement for a *Saccharomyces cerevisiae* protein kinase C homolog. Mol Cell Biol. 1992;12(1):172–82. 10.1128/mcb.12.1.172 1729597 PMC364081

[14] Xu JR, Staiger CJ, Hamer JE. Inactivation of the mitogen-activated protein kinase Mps1 from the rice blast fungus prevents penetration of host cells but allows activation of plant defense responses. Proc Natl Acad Sci U S A. 1998;95(21):12713–8. 10.1073/pnas.95.21.12713 9770551 PMC22896

[15] Jeon J, Goh J, Yoo S, Chi MH, Choi J, Rho HS, et al A putative MAP kinase kinase kinase, MCK1, is required for cell wall integrity and pathogenicity of the rice blast fungus, *Magnaporthe oryzae*. Mol Plant Microbe Interact. 2008;21(5):525–34. 10.1094/MPMI-21-5-0525 18393612

[16] Yin Z, Feng W, Chen C, Xu J, Li Y, Yang L, et al Shedding light on autophagy coordinating with cell wall integrity signaling to govern pathogenicity of *Magnaporthe oryzae*. Autophagy. 2020;16(5):900–16. 10.1080/15548627.2019.1644075 31313634 PMC7144863

[17] Hamer JE, Howard RJ, Chumley FG, Valent B. A mechanism for surface attachment in spores of a plant pathogenic fungus. Science. 1988;239(4837):288–90. 10.1126/science.239.4837.288 17769992

[18] Ryder LS, Talbot NJ. Regulation of appressorium development in pathogenic fungi. Curr Opin Plant Biol. 2015;26:8–13. 10.1016/j.pbi.2015.05.013 26043436 PMC4781897

[19] Fernandez J, Orth K. Rise of a Cereal Killer: The Biology of *Magnaporthe oryzae* Biotrophic Growth. Trends Microbiol. 2018;26(7):582–97. 10.1016/j.tim.2017.12.007 29395728 PMC6003838

[20] Jelitto TC, Page HA, Read ND. Role of external signals in regulating the pre-penetration phase of infection by the rice blast fungus, *Magnaporthe grisea*. Planta. 1995;194(4):471–7.

[21] Liu H, Suresh A, Willard FS, Siderovski DP, Lu S, Naqvi NI. Rgs1 regulates multiple Galpha subunits in *Magnaporthe* pathogenesis, asexual growth and thigmotropism. EMBO J. 2007;26(3):690–700. 10.1038/sj.emboj.7601536 17255942 PMC1794393

[22] Fukada F, Kodama S, Nishiuchi T, Kajikawa N, Kubo Y. Plant pathogenic fungi *Colletotrichum* and *Magnaporthe* share a common G(1) phase monitoring strategy for proper appressorium development. New Phytol. 2019;222(4):1909–23. 10.1111/nph.15728 30715740

[23] Saunders DG, Dagdas YF, Talbot NJ. Spatial uncoupling of mitosis and cytokinesis during appressorium-mediated plant infection by the rice blast fungus *Magnaporthe oryzae*. Plant Cell. 2010;22(7):2417–28. 10.1105/tpc.110.074492 20639448 PMC2929119

[24] Veneault-Fourrey C, Barooah M, Egan M, Wakley G, Talbot NJ. Autophagic fungal cell death is necessary for infection by the rice blast fungus. Science. 2006;312(5773):580–3. 10.1126/science.1124550 16645096

[25] Veneault-Fourrey C, Talbot NJ. Autophagic cell death and its importance for fungal developmental biology and pathogenesis. Autophagy. 2007;3(2):126–7. 10.4161/auto.3529 17172805

[26] Kershaw MJ, Talbot NJ. Genome-wide functional analysis reveals that infection-associated fungal autophagy is necessary for rice blast disease. Proc Natl Acad Sci U S A. 2009;106(37):15967–72. 10.1073/pnas.0901477106 19717456 PMC2747227

[27] Yin Z, Chen C, Yang J, Feng W, Liu X, Zuo R, et al Histone acetyltransferase MoHat1 acetylates autophagy-related proteins MoAtg3 and MoAtg9 to orchestrate functional appressorium formation and pathogenicity in *Magnaporthe oryzae*. Autophagy. 2019;15(7):1234–57. 10.1080/15548627.2019.1580104 30776962 PMC6613890

[28] Scarfone I, Piatti S. Coupling spindle position with mitotic exit in budding yeast: The multifaceted role of the small GTPase Tem1. Small GTPases. 2015;6(4):196–201. 10.1080/21541248.2015.1109023 26507466 PMC4905282

[29] Rock JM, Amon A. Cdc15 integrates Tem1 GTPase-mediated spatial signals with Polo kinase-mediated temporal cues to activate mitotic exit. Genes Dev. 2011;25(18):1943–54. 10.1101/gad.17257711 21937712 PMC3185966

[30] Davì V, Tanimoto H, Ershov D, Haupt A, De Belly H, Le Borgne R, et al Mechanosensation dynamically coordinates polar growth and cell wall assembly to promote cell survival. Dev Cell. 2018;45(2):170–82.e7. 10.1016/j.devcel.2018.03.022 29689193

[31] Sablowski R. Coordination of plant cell growth and division: collective control or mutual agreement? Curr Opin Plant Biol. 2016;34:54–60. 10.1016/j.pbi.2016.09.004 27723536

[32] Sampathkumar A, Peaucelle A, Fujita M, Schuster C, Persson S, Wasteneys GO, et al Primary wall cellulose synthase regulates shoot apical meristem mechanics and growth. Development. 2019;146(10). 10.1242/dev.179036 31076488 PMC6550022

[33] Lee SE, Frenz LM, Wells NJ, Johnson AL, Johnston LH. Order of function of the budding-yeast mitotic exit-network proteins Tem1, Cdc15, Mob1, Dbf2, and Cdc5. Curr Biol. 2001;11(10):784–8. 10.1016/s0960-9822(01)00228-7 11378390

[34] Qian B, Liu X, Jia J, Cai Y, Chen C, Zhang H, et al MoPpe1 partners with MoSap1 to mediate TOR and cell wall integrity signalling in growth and pathogenicity of the rice blast fungus *Magnaporthe oryzae*. Environ Microbiol. 2018;20(11):3964–79. 10.1111/1462-2920.14421 30246284 PMC6790007

[35] Liu X, Cai Y, Zhang X, Zhang H, Zheng X, Zhang Z. Carbamoyl phosphate synthetase subunit MoCpa2 affects development and pathogenicity by modulating arginine biosynthesis in *Magnaporthe oryzae*. Front Microbiol. 2016;7:2023 10.3389/fmicb.2016.02023 28066349 PMC5166579

[36] Thines E, Weber RW, Talbot NJ. MAP kinase and protein kinase A-dependent mobilization of triacylglycerol and glycogen during appressorium turgor generation by *Magnaporthe grisea*. Plant Cell. 2000;12(9):1703–18. 10.1105/tpc.12.9.1703 11006342 PMC149080

[37] Zhang H, Liu K, Zhang X, Song W, Zhao Q, Dong Y, et al A two-component histidine kinase, MoSLN1, is required for cell wall integrity and pathogenicity of the rice blast fungus, *Magnaporthe oryzae*. Curr Genet. 2010;56(6):517–28. 10.1007/s00294-010-0319-x 20848286

[38] Zhang H, Tang W, Liu K, Huang Q, Zhang X, Yan X, et al Correction: Eight RGS and RGS-like proteins orchestrate growth, differentiation, and pathogenicity of *Magnaporthe oryzae*. PLoS Pathog. 2019;15(11):e1008187 10.1371/journal.ppat.1008187 31730634 PMC6857851

[39] Watanabe H, Azuma M, Igarashi K, Ooshima H. Analysis of chitin at the hyphal tip of *Candida albicans* using calcofluor white. Biosci Biotechnol Biochem. 2005;69(9):1798–801. 10.1271/bbb.69.1798 16195606

[40] Nakajima T, Tamari K, Matsuda K. A cell wall proteo-heteroglycan from *Piricularia oryzae*: isolation and partial structure. J Biochem. 1977;82(6):1647–55. 10.1093/oxfordjournals.jbchem.a131860 563863

[41] Wolf S. Plant cell wall signalling and receptor-like kinases. Biochem J. 2017;474(4):471–92. 10.1042/BCJ20160238 28159895

[42] Abenza JF, Couturier E, Dodgson J, Dickmann J, Chessel A, Dumais J, et al Wall mechanics and exocytosis define the shape of growth domains in fission yeast. Nat Commun. 2015;6:8400 10.1038/ncomms9400 26455310 PMC4618311

[43] Engelsdorf T, Gigli-Bisceglia N, Veerabagu M, McKenna JF, Vaahtera L, Augstein F, et al The plant cell wall integrity maintenance and immune signaling systems cooperate to control stress responses in *Arabidopsis thaliana*. Sci Signal. 2018;11(536). 10.1126/scisignal.aao3070 29945884

[44] Banavar SP, Gomez C, Trogdon M, Petzold LR, Yi TM, Campàs O. Mechanical feedback coordinates cell wall expansion and assembly in yeast mating morphogenesis. PLoS Comput Biol. 2018;14(1):e1005940 10.1371/journal.pcbi.1005940 29346368 PMC5790295

[45] Hartwell L, Kastan M. Cell cycle control and cancer. Science. 1988;266(5192):1821–8. 10.1126/science.7997877 7997877

[46] Vermeulen K, Van Bockstaele DR, Berneman ZN. The cell cycle: a review of regulation, deregulation and therapeutic targets in cancer. Cell Prolif. 2003;36(3):131–49. 10.1046/j.1365-2184.2003.00266.x 12814430 PMC6496723

[47] Hall MC. Proteomics modifies our understanding of cell cycle complexity. Sci Signal. 2010;3(106):pe4 10.1126/scisignal.3106pe4 20103771

[48] Gladfelter AS, Kozubowski L, Zyla TR, Lew DJ. Interplay between septin organization, cell cycle and cell shape in yeast. J Cell Sci. 2005;118(Pt 8):1617–28. 10.1242/jcs.02286 15784684

[49] Kono K, Al-Zain A, Schroeder L, Nakanishi M, Ikui AE. Plasma membrane/cell wall perturbation activates a novel cell cycle checkpoint during G1 in *Saccharomyces cerevisiae*. Proc Natl Acad Sci U S A. 2016;113(25):6910–5. 10.1073/pnas.1523824113 27274080 PMC4922162

[50] Yang L, Ru Y, Cai X, Yin Z, Liu X, Xiao Y, et al MoImd4 mediates crosstalk between MoPdeH-cAMP signalling and purine metabolism to govern growth and pathogenicity in *Magnaporthe oryzae*. Mol Plant Pathol. 2019;20(4):500–18. 10.1111/mpp.12770 30426699 PMC6422694

[51] Wang J, Yin Z, Tang W, Cai X, Gao C, Zhang H, et al The thioredoxin MoTrx2 protein mediates reactive oxygen species (ROS) balance and controls pathogenicity as a target of the transcription factor MoAP1 in *Magnaporthe oryzae*. Mol Plant Pathol. 2017;18(9):1199–209. Epub 2016/08/26. 10.1111/mpp.12484 27560036 PMC6638232

