# Supplementary figures and images for: Balancing of the mitotic exit network and cell wall integrity signaling governs the development and pathogenicity in Magnaporthe oryzae
Source: PLoS Pathog. 2021 Jan 7;17(1):e1009080. doi: 10.1371/journal.ppat.1009080 (PMC7817018; doi:10.1371/journal.ppat.1009080)

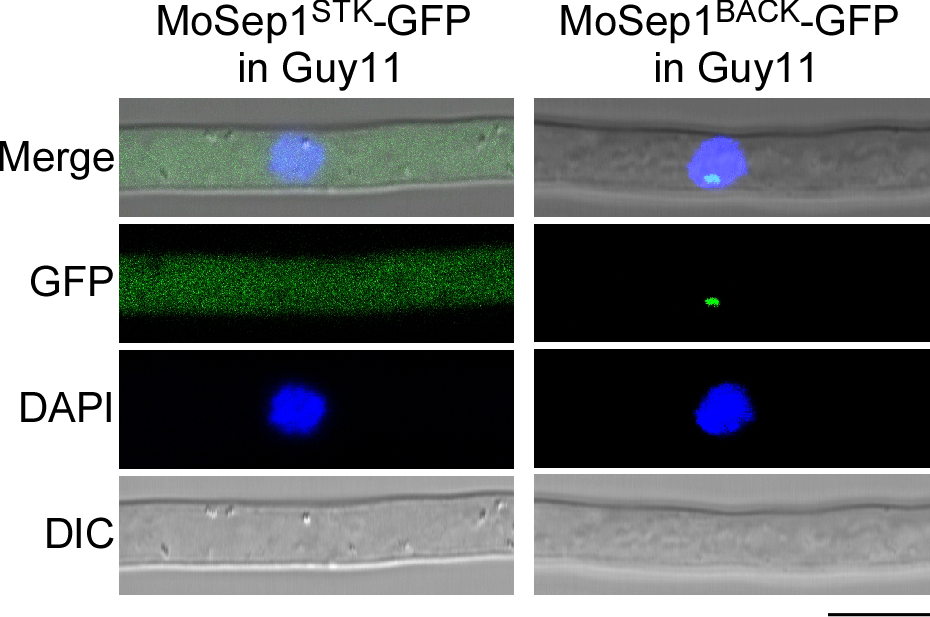

Supplement: S2 Fig — Hyphae of transformants expressing the MoSep1STK-GFP and MoSep1BACK-GFP constructs in Guy11 respectively were stained with DAPI and examined by epifluorescence microscopy. Bar, 10 μm. (TIF) [file ppat.1009080.s003.tif]

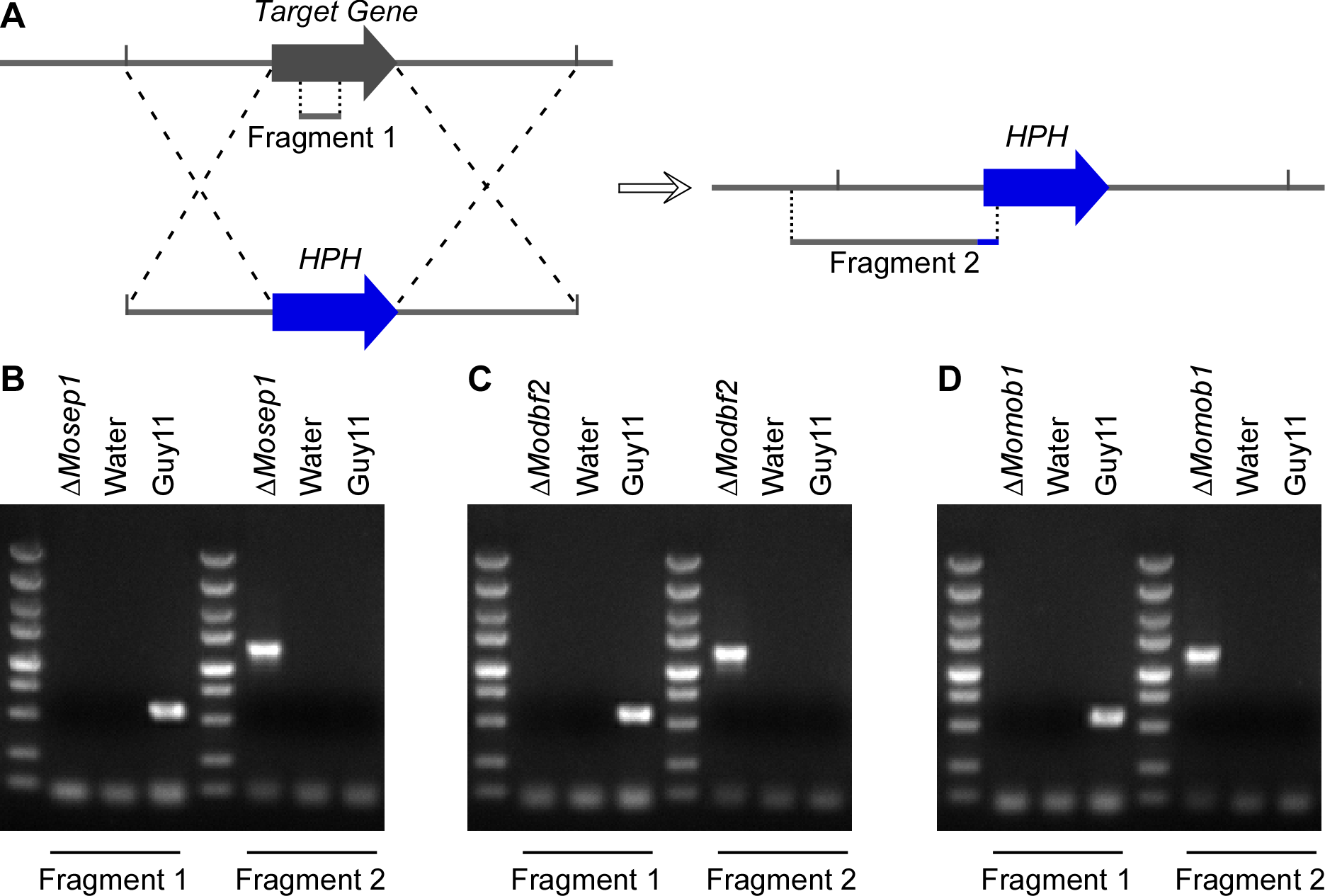

Supplement: S3 Fig — (A) Strategy of knocking out target gene in M. oryzae genome. The targe region was replaced with a 1.4-kb fragment containing the hygromycin B-resistance cassette (HPH) to create the mutant. The lines below the arrows indicated the verified fragments of the mutant. (B) PCR was used to validate the deletion of the MoSEP1 gene. When verified by fragment 1 (a 0.5-kb fragment within MoSEP1), a 0.5-kb band was observed in the wild type but not in the ΔMosep1 mutant. When verified by fragment 2 (About 1.2-kb upstream fragment of the MoMOB1 gene plus 0.1-kb fragment of HPH), the ΔMosep1 mutant (but not the wild-type) exhibited a 1.3-kb band characteristic of the gene replacement event. (C) PCR was used to validate the deletion of the MoDBF2 gene. When verified by fragment 1 (a 0.5-kb fragment within MoDBF2), a 0.5-kb band was observed in the wild type but not in the ΔModbf2 mutant. When verified by fragment 2 (About 1.2-kb upstream fragment of the MoMOB1 gene plus 0.1-kb fragment of HPH), the ΔModbf2 mutant (but not the wild-type) exhibited a 1.3-kb band characteristic of the gene replacement event. (D) PCR was used to validate the deletion of the MoMOB1 gene. When verified by fragment 1 (a 0.5-kb fragment within MoMOB1), a 0.5-kb band was observed in the wild type but not in the ΔMomob1 mutant. When verified by fragment 2 (About 1.2-kb upstream fragment of the MoMOB1 gene plus 0.1-kb fragment of HPH), the ΔMomob1 mutant (but not the wild-type) exhibited a 1.3-kb band characteristic of the gene replacement event. (TIF) [file ppat.1009080.s004.tif]

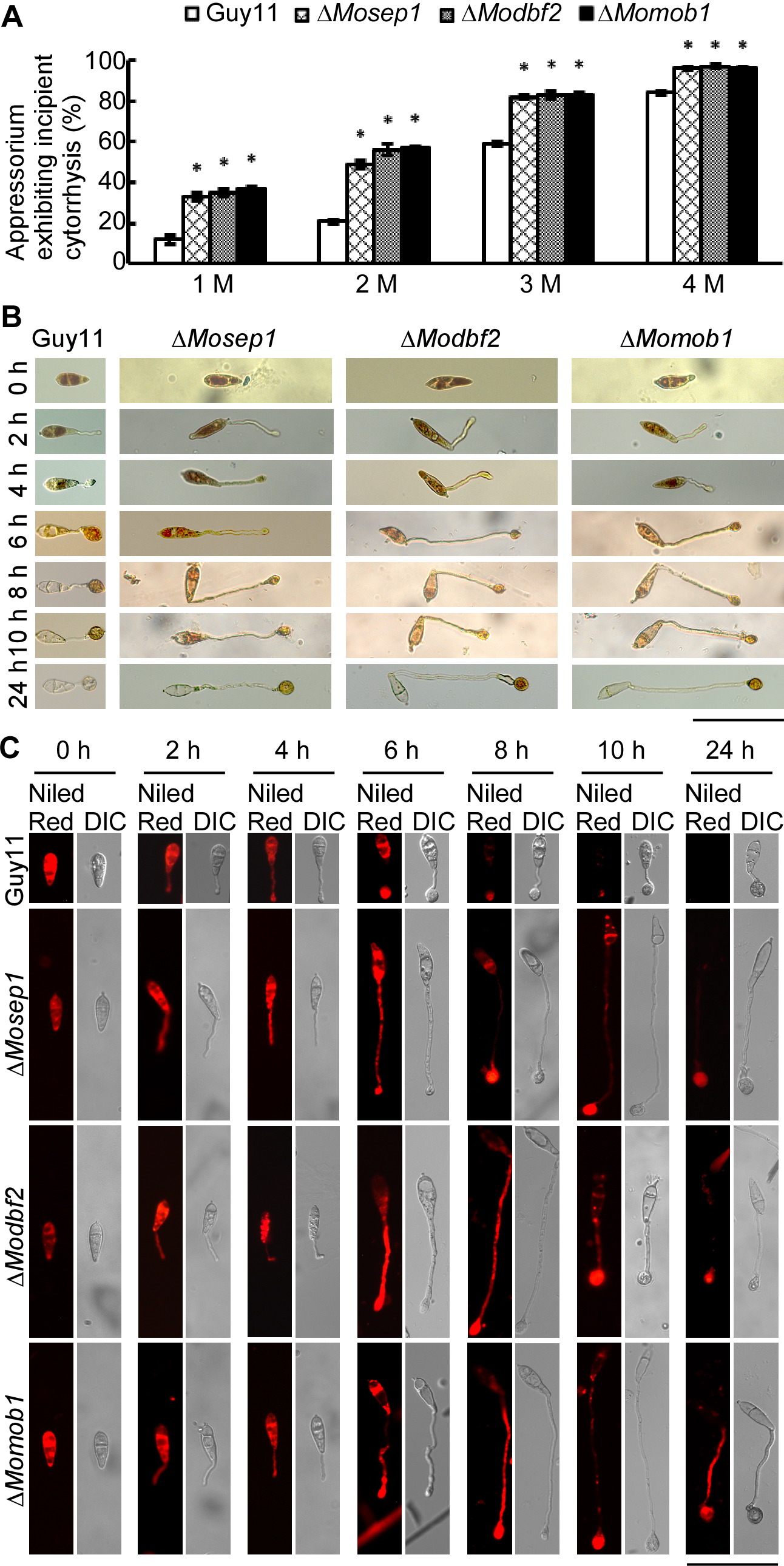

Supplement: S4 Fig — (A) Conidia were incubated on the hydrophobic surface. The cytorrhysis assay using various concentrations of glycerol (1–4 M). For each glycerol concentration, at least 100 appressoria were observed and the number of collapsed appressoria was counted from three independent experiments. Error bars represented the standard deviations. Asterisks denote statistical significance according to a Student’s test (p<0.01). (B) Conidia were incubated on the hydrophobic surface. Samples were stained with I2/KI solution at different time points and yellowish-brown glycogen deposits became visible immediately. Bar, 50 μm. (C) Conidia were incubated on the hydrophobic surface. Samples were stained for the presence of lipid bodied by Nile red. Bar, 50 μm. (TIF) [file ppat.1009080.s005.tif]

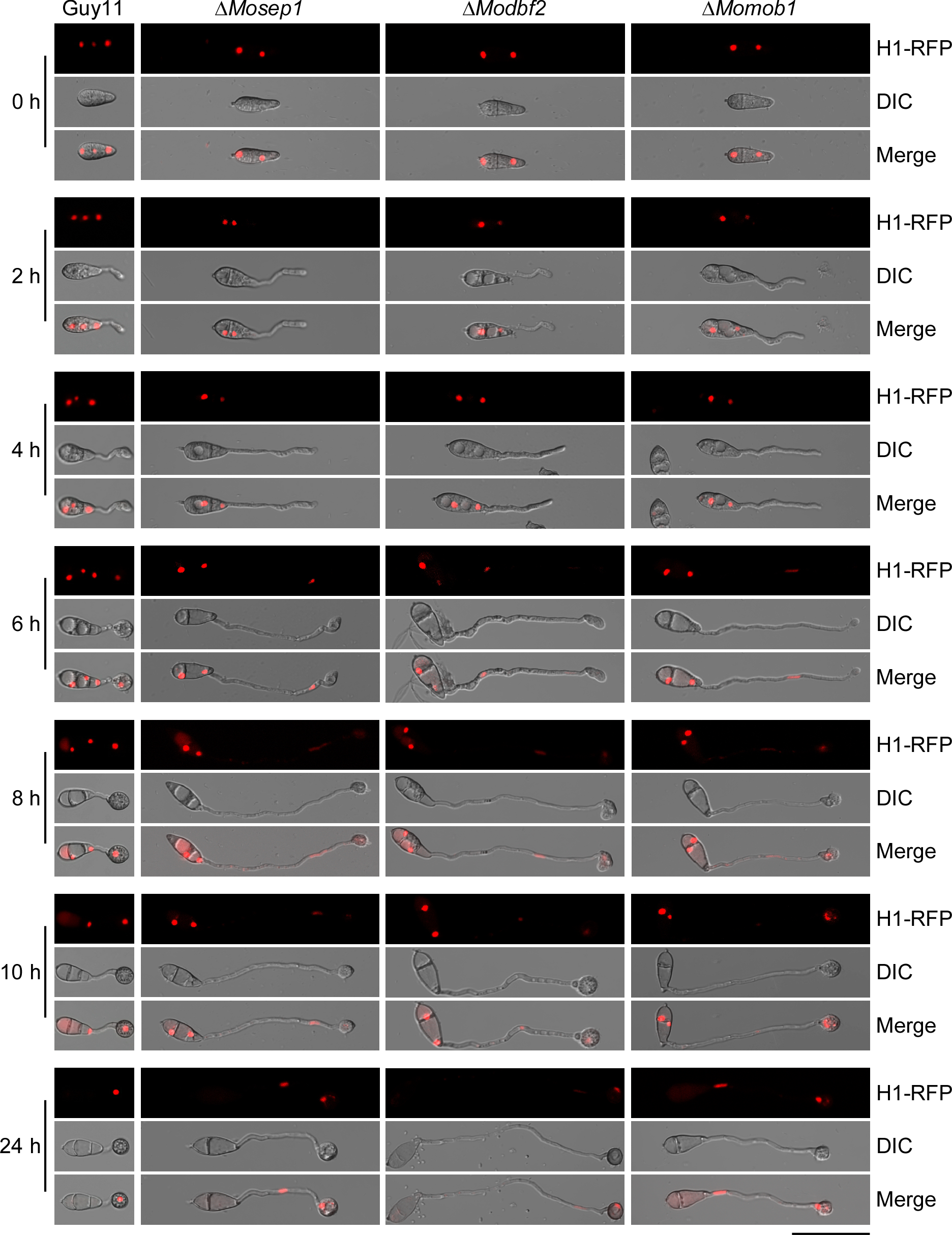

Supplement: S5 Fig — Conidia were allowed to germinate on the hydrophobic surface. Conidia germination of transformants of Guy11 and the ΔMosep1, ΔModbf2, ΔMomob1 mutants expressing the H1-RFP construct were stained with CFW at different time points and examined by epifluorescence microscopy. Bar, 50 μm. (TIF) [file ppat.1009080.s006.tif]

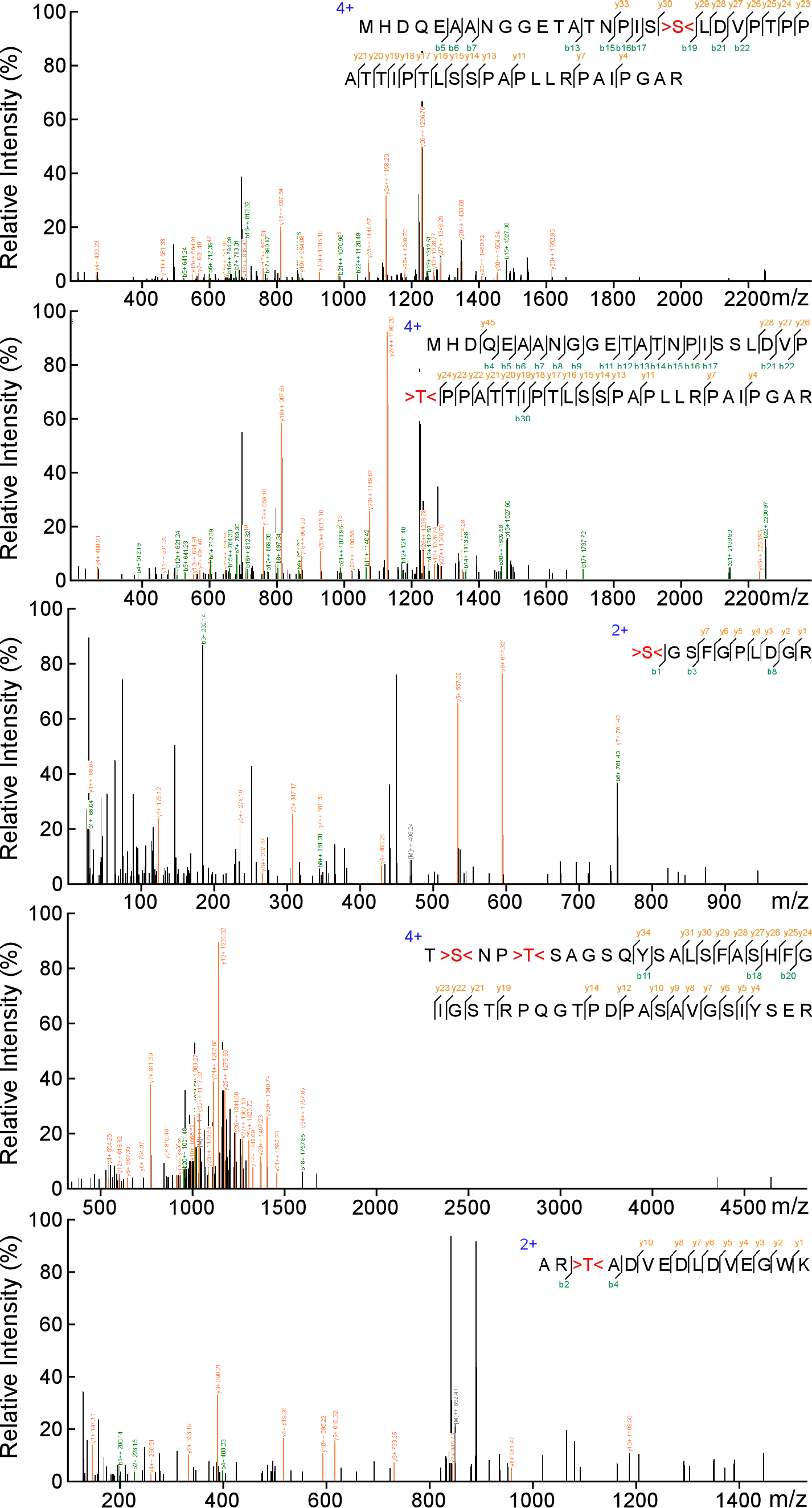

Supplement: S7 Fig — MoMkk1 phosphorylation sites in the Guy11 compared with the ΔMosep1 mutant expressing MoMKK1 was identified by MS analysis. (TIF) [file ppat.1009080.s008.tif]

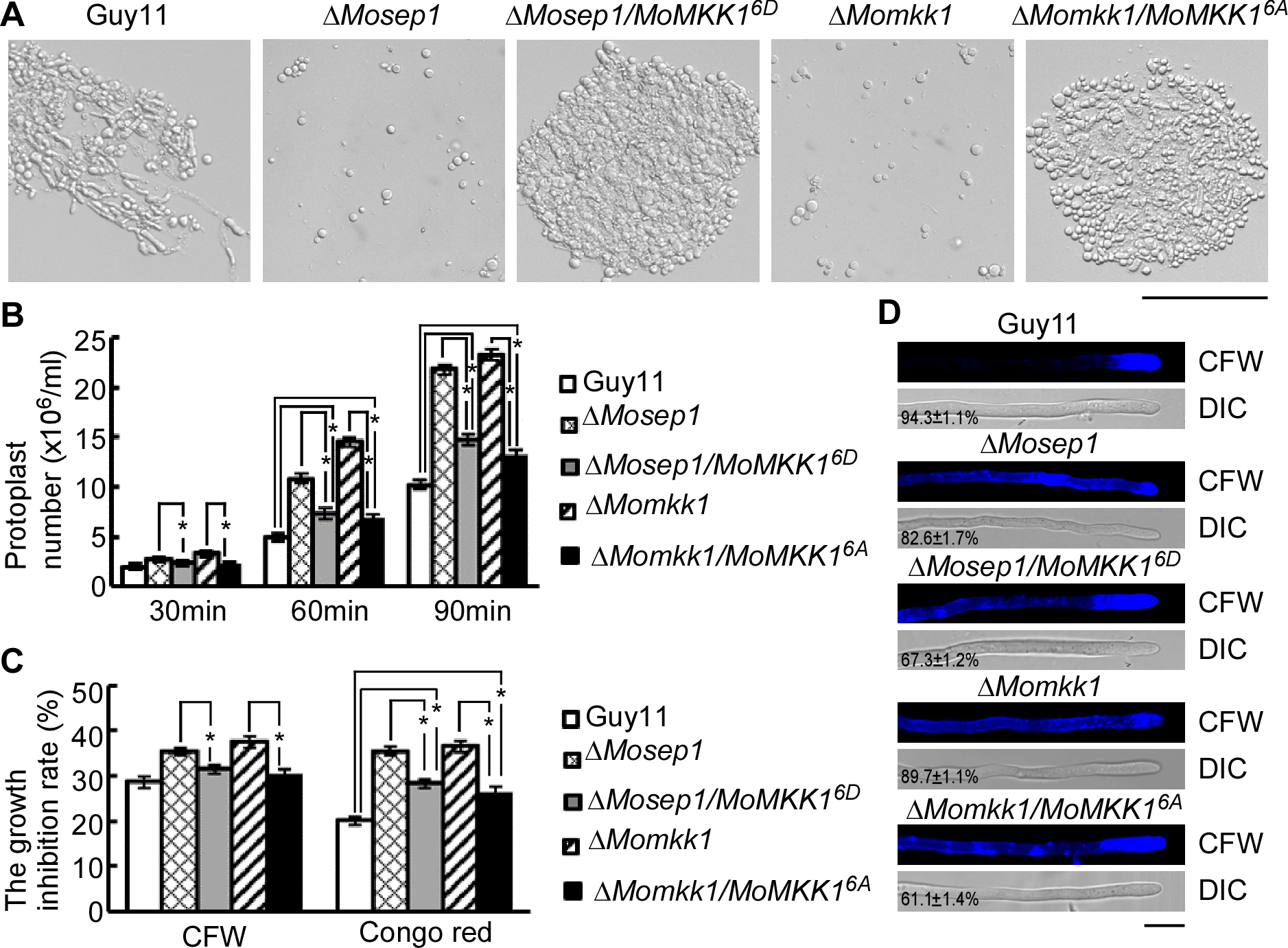

Supplement: S9 Fig — (A) Light microscopic examination of protoplast release of Guy11, the ΔMosep1, ΔMosep1/MoMKK16D, ΔMomkk1, ΔMomkk1/MoMKK16A mutants after treatment with cell wall-degrading enzymes for 45 min at 30°C. Bar, 50 μm (B) Statistical analysis of protoplast release of Guy11, the ΔMosep1, ΔMosep1/MoMKK16D, ΔMomkk1, ΔMomkk1/MoMKK16A mutants after treatment with cell wall-degrading enzymes for 30 min, 60 min, and 90 min at 30°C. Error bars represented the standard deviations from three independent experiments. Asterisks denote statistical significance according to a Student’s test (p<0.01). (C) The hyphal diameter of Guy11, the ΔMosep1, ΔMosep1/MoMKK16D, ΔMomkk1, ΔMomkk1/MoMKK16A mutants examined 7 days after incubation on CM agar plates with cell wall-perturbing agents; 400 μg/ml for CFW, and 400 μg/ml for Congo red. The experiments were repeated three times. Error bars represented the standard deviations. Asterisks denote statistical significance according to a Student’s test (p<0.01). (D) Hyphae of Guy11, the ΔMosep1, ΔMosep1/MoMKK16D, ΔMomkk1, ΔMomkk1/MoMKK16A mutants were stained with CFW for 5 min in the darkness. The distribution of chitin in the cell wall was disrupted. (TIF) [file ppat.1009080.s010.tif]

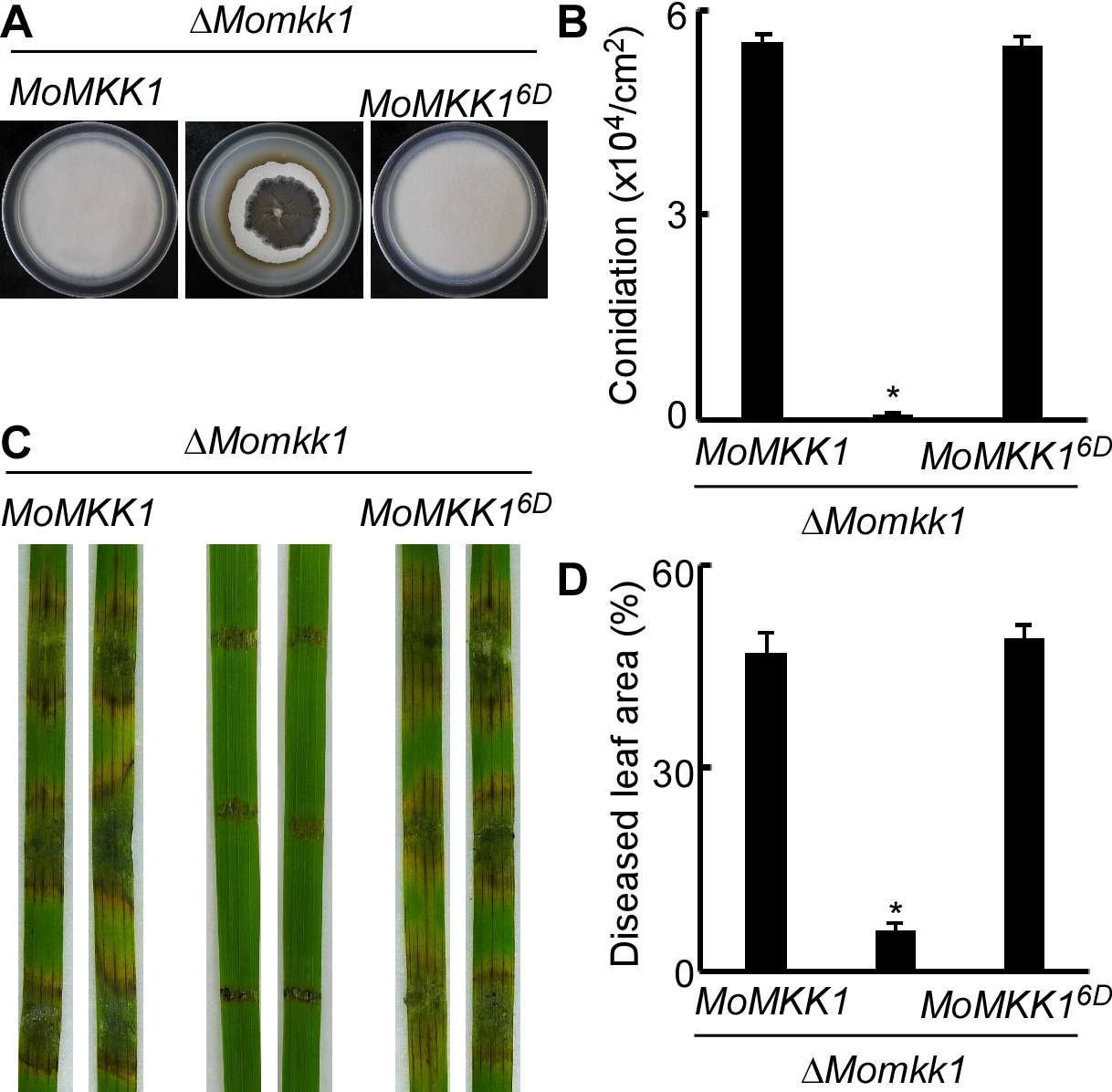

Supplement: S10 Fig — (A) Autolysis observation of ΔMomkk1/MoMKK1, ΔMomkk1, ΔMomkk1/MoMKK16D (ΔMomkk1/MoMKK1S19D, T24D, S125D, S136D, T139D, T207D), mutant strains on CM medium. (B) Statistical analysis of conidia production on SDC medium cultured at 28°C for 7 days in the dark followed by 3 days of continuous illumination under fluorescent light. Error bars represent the standard deviations from three independent experiments. Asterisks indicate statistical significance according to a Student’s test (p<0.01). (C) Pathogenicity test on rice leaves. Wounded rice leaves were incubated with different strains. Diseased leaves were photographed 4 days after inoculation. (D) Diseased leaf area analysis of (C). Data were present as a bar chart showing the percentage of lesion areas. Error bars represented the standard deviations from three independent experiments. Asterisks indicate statistical significance according to a Student’s test (p<0.01). (TIF) [file ppat.1009080.s011.tif]
